# Toward AI-Assisted Sickle Cell Screening: A Controlled Comparison of CNN, Transformer, and Hybrid Architectures Using Public Blood-Smear Images

**DOI:** 10.3390/diagnostics16030414

**Published:** 2026-01-29

**Authors:** Linah Tasji, Hanan S. Alghamdi, Abdullah S Almalaise Al-Ghamdi

**Affiliations:** 1 Department of Information Systems, Faculty of Computing and Information Technology, King Abdulaziz University, Jeddah 21589, Saudi Arabia; ltasji@stu.kau.edu.sa (L.T.); aalmalaise@kau.edu.sa (A.S.A.A.-G.); 2 Blood Disorders Administration, Ministry of Health, Riyadh 11176, Saudi Arabia; 3 Computer Science Department, School of Engineering, Computing and Design, Dar Al-Hekma University, Jeddah 22246, Saudi Arabia

**Keywords:** vision transformers (ViTs), convolutional neural networks (CNNs), sickle cell disease (SCD), blood smear images, peripheral blood smear (PBS), deep learning, robust evaluation

## Abstract

**Background**: Sickle cell disease (SCD) is a prevalent hereditary hemoglobinopathy associated with substantial morbidity, particularly in regions with limited access to advanced laboratory diagnostics. Conventional diagnostic workflows, including manual peripheral blood smear examination and biochemical or molecular assays, are resource-intensive, time-consuming, and subject to observer variability. Recent advances in artificial intelligence (AI) enable automated analysis of blood smear images and offer a scalable alternative for SCD screening. **Methods**: This study presents a controlled benchmark of CNNs, Vision Transformers, hierarchical Transformers, and hybrid CNN–Transformer architectures for image-level SCD classification using a publicly available peripheral blood smear dataset. Eleven ImageNet-pretrained models were fine-tuned under identical conditions using an explicit leakage-safe evaluation protocol, incorporating duplicate-aware, group-based data splitting and repeated splits to assess robustness. Performance was evaluated using accuracy and macro-averaged precision, recall, and F1-score, complemented by bootstrap confidence intervals, paired statistical testing, error-type analysis, and explainable AI (XAI). **Results**: Across repeated group-aware splits, CNN-based and hybrid architectures demonstrated more stable and consistently higher performance than transformer-only models. MaxViT-Tiny and DenseNet121 ranked highest overall, while pure ViTs showed reduced effectiveness under data-constrained conditions. Error analysis revealed a dominance of false-positive predictions, reflecting intrinsic morphological ambiguity in challenging samples. XAI visualizations suggest that CNNs focus on localized red blood cell morphology, whereas hybrid models integrate both local and contextual cues. **Conclusions**: Under limited-data conditions, convolutional inductive bias remains critical for robust blood-smear-based SCD classification. CNN and hybrid CNN–Transformer models offer interpretable and reliable performance, supporting their potential role as decision-support tools in screening-oriented research settings.

## 1. Introduction

Sickle cell disease (SCD) is the most prevalent inherited hemoglobinopathy worldwide and represents a major public health challenge, with approximately 300,000 affected newborns reported annually [1]. The disease disproportionately affects regions with historical malaria endemicity, including sub-Saharan Africa, the Mediterranean basin, and the Middle East. However, in Saudi Arabia, SCD and sickle cell trait (SCT) remain highly prevalent, particularly in the Eastern and Southern regions, where carrier rates are among the highest globally [2,3,4]. This geographic distribution is largely attributed to the protective effect of SCT against severe malaria, a phenomenon widely referred to as the malaria hypothesis [1].

Despite the implementation of nationwide premarital and newborn screening programs in Saudi Arabia, SCD continues to pose substantial diagnostic and management challenges. National screening initiatives have consistently identified a high proportion of asymptomatic SCT carriers, underscoring the ongoing need for effective early detection, risk stratification, and scalable diagnostic solutions [2,3,4,5]. These challenges are further compounded by the marked clinical heterogeneity of SCD, which ranges from mild anemia to severe vaso-occlusive and hemolytic complications, complicating timely diagnosis and clinical decision-making in routine healthcare settings.

Accurate diagnosis of SCD is inherently challenging due to significant variability in red blood cell (RBC) morphology, low concentrations of sickled cells in peripheral blood samples, and the subjectivity associated with manual blood smear interpretation. Conventional diagnostic workflows rely on peripheral blood smear examination and screening assays such as the solubility test, followed by confirmatory biochemical or molecular techniques, including high-performance liquid chromatography (HPLC) and polymerase chain reaction (PCR) [6]. While these methods provide high diagnostic accuracy and genotype-specific information, they are resource-intensive, time-consuming, and require specialized laboratory infrastructure, limiting their accessibility in peripheral or resource-limited healthcare settings [6,7].

In recent years, artificial intelligence (AI) has emerged as a promising approach to augment hematological diagnostics through automated analysis of blood smear images. Deep learning models, particularly convolutional neural networks (CNNs), have demonstrated strong performance in detecting sickled RBCs in controlled experimental settings, primarily due to their inductive bias toward localized texture, contour, and shape features [8,9,10]. In parallel, portable point-of-care technologies such as HemoTypeSC and shape-based hypoxia assays have demonstrated high diagnostic accuracy, supporting the feasibility of rapid and scalable screening strategies for SCD, particularly in resource-limited environments [7,11,12].

Beyond CNNs, transformer-based architectures—most notably Vision Transformers (ViTs)—have gained increasing attention in medical image analysis due to their ability to model long-range dependencies and global contextual relationships through self-attention mechanisms [13,14,15,16]. ViTs have demonstrated competitive performance in several medical imaging applications, particularly when sufficient training data are available [14,16]. However, their reliance on global self-attention and lack of convolutional inductive bias may limit effectiveness in data-constrained hematological imaging tasks characterized by subtle local morphological features [13,15].

To address these limitations, hierarchical and hybrid CNN–Transformer architectures have been proposed. Hierarchical transformers, such as the Swin Transformer, introduce shifted-window self-attention to enhance local context modeling while maintaining computational efficiency [17]. Hybrid architectures, including MaxViT, explicitly integrate convolutional feature extraction with multi-axis self-attention, enabling simultaneous learning of fine-grained local morphology and global contextual relationships [18]. Such designs have demonstrated strong performance across medical imaging benchmarks, particularly under data-constrained conditions.

Despite these advances, systematic evaluations comparing CNNs, pure ViTs, hierarchical transformers, and hybrid architectures for SCD classification from peripheral blood smear images remain limited. In particular, there is a lack of consensus regarding the relative suitability of these architectural paradigms for hematological image analysis, where diagnostically relevant information is predominantly encoded in subtle local morphological variations rather than global spatial context.

This study addresses this gap by conducting a controlled, leakage-safe comparative evaluation of CNN, transformer, and hybrid deep learning architectures for image-level SCD classification using peripheral blood smear images. The evaluation emphasizes robustness through repeated group-aware data splits and focuses on understanding how architectural inductive bias influences performance under data-constrained conditions, rather than claiming universal model superiority. From a national perspective, this work aligns with Saudi Arabia’s Vision 2030 and the Health Sector Transformation Program, which emphasize digital health innovation and the integration of AI-driven decision-support tools to support early disease detection within clinical workflows [19,20]. Ultimately, this research seeks to support the development of scalable, AI-assisted diagnostic tools that can complement existing screening programs and strengthen SCD diagnostic pathways in Saudi Arabia and the wider Middle East.

Accordingly, all conclusions are interpreted within the scope of the dataset, experimental design, and statistical uncertainty.

This study contributes a controlled and leakage-safe benchmark for sickle cell disease classification from peripheral blood smear images. Specifically, it provides a unified comparison of 11 ImageNet-pretrained CNN, transformer, and hybrid CNN–Transformer architectures under identical preprocessing, training budgets, and evaluation conditions, enabling isolation of architectural inductive bias under data-constrained settings. The evaluation adopts a duplicate-aware, group-based splitting strategy with repeated runs to prevent information leakage and assess robustness. Beyond point estimates, the analysis integrates statistical uncertainty, structured error-type analysis highlighting false-positive dominance in screening contexts, and calibration-aware evaluation. Finally, qualitative explainability analysis compares attention patterns across architectural families, offering interpretability insights into how CNNs, transformers, and hybrid models differ in focusing on red blood cell morphology.

## 2. Related Works

### 2.1. Automated Blood Smear Analysis for SCD

Deep learning approaches for blood smear analysis have demonstrated strong performance in morphology-driven tasks, particularly when convolutional inductive bias aligns with localized cellular features [21]. Automated analysis of peripheral blood smear images has therefore been widely explored as a means to reduce subjectivity and improve scalability in sickle cell disease (SCD) diagnosis. Early approaches relied on classical image processing and machine learning techniques, where handcrafted morphological features were extracted from segmented red blood cells and classified using conventional classifiers. For example, Das et al. employed region-based segmentation methods followed by support vector machines, achieving up to 95% accuracy in distinguishing healthy and sickled cells [7]. While effective under controlled conditions, such methods exhibited limited robustness to variability in staining, illumination, and cell morphology.

Deep learning methods have since become the dominant paradigm due to their ability to learn hierarchical feature representations directly from raw images. Convolutional neural networks (CNNs), in particular, have demonstrated strong performance in detecting sickled cells by leveraging their inductive bias toward localized texture and shape features. Goswami et al. applied transfer learning using pretrained CNN architectures, including GoogLeNet and ResNet variants, achieving an accuracy of 94.9% for SCD classification [8,22]. Similarly, weakly supervised frameworks have been proposed to mitigate the cost of detailed annotations. Manescu et al. introduced the Multiple Objects Feature Fusion (MOFF) approach, which aggregates CNN features across multiple fields of view without object-level labels, achieving 91% accuracy for SCD detection [9]. Although these methods substantially reduce annotation burden, they remain largely limited to binary classification tasks and single-center datasets.

To further enhance scalability and interpretability, attention-based multiple instance learning (MIL) models have been explored. Sadafi et al. proposed an attention-driven MIL framework that integrates a pretrained region-based CNN with attention mechanisms to classify genetic blood disorders, including SCD, without requiring cell-level annotations [10]. While this approach improves interpretability and reduces annotation requirements, its performance remains constrained, and the authors emphasized the need for richer morphological modeling and uncertainty-aware predictions to support clinical deployment.

Beyond image-only models, multimodal and point-of-care diagnostic strategies have also been investigated. Gedefaw et al. combined CNN-based blood smear analysis with neural network-based genomic data integration, achieving high diagnostic accuracy for hematologic disorders [6]. In parallel, shape-based point-of-care assays such as ShapeDx™ demonstrated full concordance with high-performance liquid chromatography (HPLC) results by quantifying red blood cell deformation under hypoxic conditions [11]. Despite their diagnostic precision, such approaches rely on specialized equipment, multimodal data, or limited validation cohorts, which may restrict scalability and broader clinical adoption.

Taken together, existing blood smear-based approaches demonstrate that high diagnostic accuracy for SCD is achievable using automated image analysis. However, most prior studies either rely on CNNs with strong local feature bias or adopt task-specific pipelines without systematically evaluating how architectural design choices influence performance under data-constrained conditions. This gap motivates the need for controlled, architecture-level comparisons under unified experimental settings.

### 2.2. CNNs, ViTs, and Hybrid Architectures in Medical Imaging

CNNs have long been the backbone of medical image analysis due to their ability to efficiently capture localized spatial features through convolution and pooling operations [21,22,23,24,25]. These characteristics make CNNs particularly well suited for morphology-driven tasks such as blood smear analysis, where diagnostically relevant information is often encoded in subtle local variations in cell shape and texture.

ViTs represent a fundamentally different modeling paradigm, replacing convolution with self-attention to capture global contextual relationships across image patches [18]. ViTs have demonstrated strong performance in several medical imaging applications, particularly in tasks requiring global feature integration and fine-grained discrimination. Systematic reviews and comparative studies have shown that ViTs can outperform CNNs in selected domains, including retinal disease detection and thoracic imaging, when sufficient training data are available [14,16].

However, ViTs lack the inherent convolutional inductive biases that enable CNNs to learn efficiently from limited data. Naseer et al. demonstrated that although ViTs exhibit robustness to occlusions, their performance advantage diminishes in data-constrained settings typical of many medical imaging tasks [15]. These observations are particularly relevant for hematological image analysis, where datasets are often small and morphological cues are highly localized.

To bridge this gap, hierarchical and hybrid CNN–Transformer architectures have been proposed. Hierarchical transformers such as the Swin Transformer introduce shifted-window self-attention to better capture local context while preserving global modeling capability [17]. Hybrid architectures, including MaxViT, explicitly integrate convolutional layers with multi-axis self-attention, enabling the model to benefit simultaneously from local feature extraction and global contextual reasoning [18]. Such designs have shown superior performance across diverse medical imaging benchmarks, particularly under limited data conditions.

Despite the growing adoption of ViTs and hybrid architectures, their relative effectiveness for blood smear-based SCD classification has not been systematically examined. Existing studies often focus on a single architectural paradigm or a narrow subset of models, making it difficult to draw principled conclusions regarding the role of architectural inductive bias in morphology-driven hematological tasks.

### 2.3. Contribution of the Present Study

Building on these thematic insights, the present study provides a comprehensive and controlled comparison of CNN-based, pure ViT, hierarchical transformer, and hybrid CNN–Transformer architectures for sickle cell disease classification using peripheral blood smear (PBS) images. All models were evaluated under identical experimental conditions, allowing the impact of architectural design on classification performance to be examined in a data-constrained setting representative of real-world hematological imaging.

Rather than focusing on individual architectures in isolation, this work presents a comparative analysis based on test-set accuracy and complementary performance metrics, illustrating how different architectural paradigms respond to the morphological characteristics of red blood cells. By situating these findings within the context of Saudi Arabia’s national screening initiatives and ongoing digital health transformation efforts [5,19,20], the study offers practical, evidence-based insights to inform model selection for scalable, AI-assisted blood smear analysis that can complement existing diagnostic workflows.

Table 1 provides a contextual comparison of prior AI-based sickle cell disease blood-smear studies, emphasizing methodological differences in data sources, supervision level, and evaluation practices rather than direct numerical performance comparison.

Direct numerical comparison across studies should be interpreted with caution due to differences in task granularity (image-level vs. sample-level), dataset composition, annotation strategies, and validation protocols. Accordingly, the table contextualizes methodological trends rather than claiming direct performance superiority.

## 3. Materials and Methods

**Figure 1:**
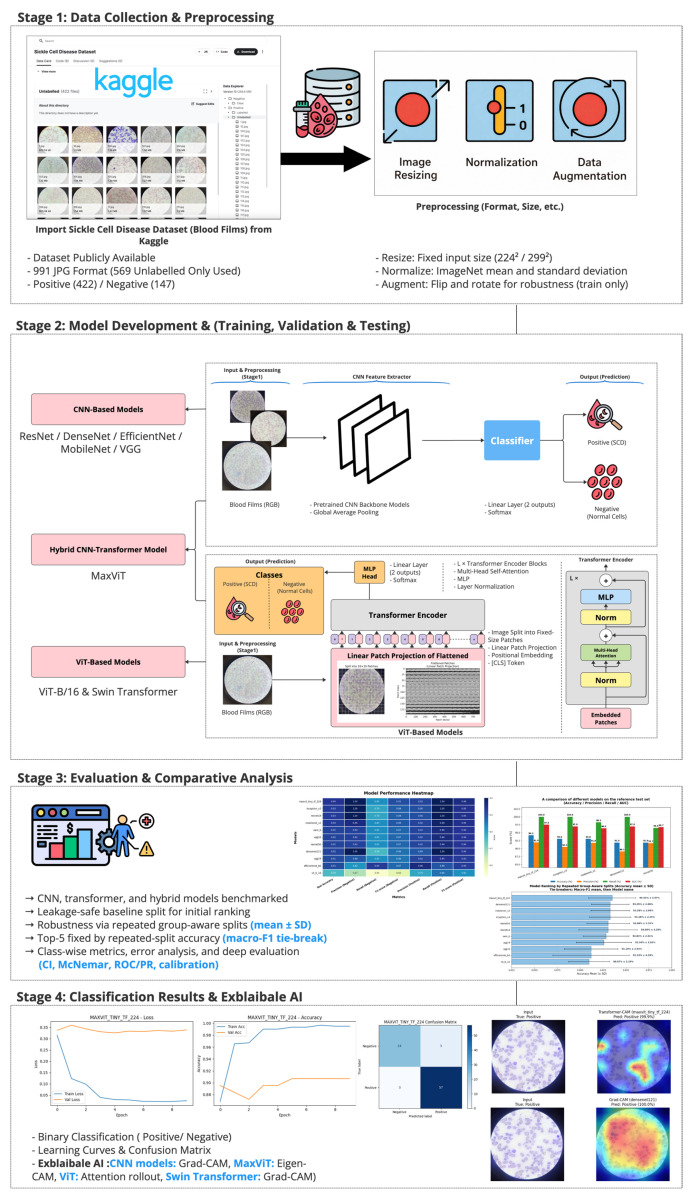
Schematic overview of the proposed experimental pipeline for automated SCD classification.

This study employed a transparent and reproducible experimental framework to develop and evaluate deep learning models for automated classification of SCD from PBS images. The methodological design was explicitly structured to enable a fair and controlled comparison between CNNs, ViTs, and hybrid CNN–Transformer architectures under identical experimental conditions. Figure 1 presents a schematic overview of the complete experimental workflow adopted in this study.

### 3.1. Dataset Source and Description

The dataset used in this study was obtained from a publicly available Kaggle repository [23]. It consists of microscopic PBS images acquired using standard bright-field light microscopy. The images were captured using mobile phones positioned at the microscope eyepiece, without additional optical accessories, and under varied camera settings, intentionally reflecting both optimal and suboptimal imaging conditions encountered in routine laboratory environments [24,25].

The dataset provides image-level labels for binary classification, categorizing images into Negative, representing non-sickled (normal) erythrocytes, and Positive, representing the presence of at least one sickled erythrocyte within the field of view. In addition to image-level labels, the original repository includes two subsets of positive images: a labelled subset containing rectangular bounding-box annotations around sickled cells, and an unlabeled subset without bounding boxes. In this study, only unlabeled positive images and clear negative images were used, and all images with explicit bounding-box annotations were excluded to avoid shortcut learning from non-biological visual cues introduced by annotation overlays. Although excluded from training to avoid shortcut learning, these annotated images could be leveraged in future work for quantitative localization or explainability evaluation [24,25].

**Figure 2:**
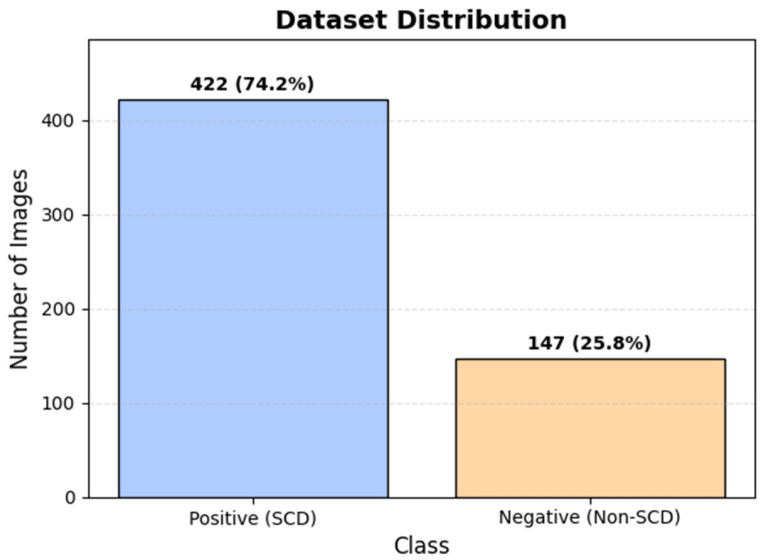
Class distribution of the clean peripheral blood smear image dataset used for sickle cell disease classification.

**Figure 3:**
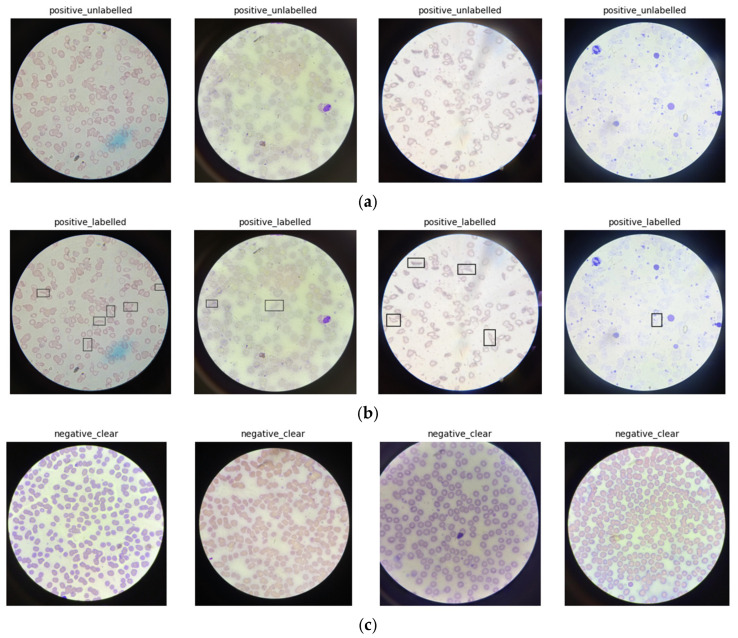
Representative PBS images from the dataset: (**a**) Positive—unlabeled images, (**b**) Positive—labeled images, where rectangular boxes indicate bounding-box annotations around sickled erythrocytes provided in the original dataset, and (**c**) Negative—unlabeled images. Annotated images were excluded from model training to avoid shortcut learning.

From the original repository, a total of 569 peripheral blood smear images were included in this benchmark study. As shown in Figure 2, the curated dataset comprises 422 SCD-positive images (74.2%) and 147 negative images (25.8%), corresponding to an approximate class imbalance ratio of 2.9:1. Figure 3 presents representative samples illustrating the morphological diversity and visual complexity of the dataset. The top row shows positive unlabeled images used for model training and evaluation, reflecting raw clinical input without annotation artifacts. The middle row presents positive labelled images with expert-annotated bounding boxes (excluded from training), and the bottom row shows negative images exhibiting normal erythrocyte morphology. Together, these examples highlight substantial variability in staining intensity, illumination, and red blood cell appearance [24,25].

The dataset includes images prepared using two staining protocols (Field and Leishman stains), different microscope models operating at ×100 objective magnification, and multiple mobile phone cameras. This heterogeneity introduces realistic variability in color distribution, contrast, and background appearance, but may also contribute to domain shifts when models are applied to images acquired under different laboratory conditions [24,25].

The source study reports that the dataset was derived from 140 blood samples collected over six months, including 105 samples from individuals with sickle cell disease and 35 from non-sicklers, with multiple images captured per smear. However, the publicly released image set does not provide patient identifiers or explicit mappings between images and individuals. Consequently, patient-level separation cannot be enforced in this benchmark, and images originating from the same individual may appear across different dataset splits. This limitation restricts direct claims of clinical generalizability and is explicitly acknowledged as a barrier to external validation [24,25].

**Scope and Data Availability Constraints.** This study is strictly limited to the use of publicly available blood smear images collected outside Saudi Arabia and released through an online repository. As such, no additional clinical metadata (e.g., laboratory values, genotype confirmation, or clinical history) or physician verification could be incorporated into the present analysis. These constraints arise from the nature of secondary analysis on publicly published datasets rather than from limitations in the experimental design. Accordingly, all analyses, results, and conclusions are interpreted solely within the scope of the released dataset and its documented acquisition conditions.

All images are fully anonymized and publicly accessible, and no personally identifiable patient information is available. Therefore, ethical approval and informed consent were not required for this secondary analysis.

A summary of the dataset characteristics, annotation types, acquisition variability, and class composition is provided in Table 2, and the overall experimental workflow is illustrated in Figure 1.

### 3.2. Data Preprocessing

All images were converted to three-channel RGB format to ensure compatibility with pretrained deep learning architectures. Images were resized to a fixed spatial resolution to provide appropriate input dimensions. Specifically, images were resized to 224 × 224 pixels for all evaluated architectures except InceptionV3, which requires a native input resolution of 299 × 299 pixels. This adjustment was made to preserve architectural integrity and ensure optimal feature extraction for each model.

Pixel intensities were normalized using ImageNet mean and standard deviation values (mean = [0.485, 0.456, 0.406], standard deviation = [0.229, 0.224, 0.225]) to enable stable fine-tuning of networks pretrained on large-scale natural image datasets. No additional image enhancement or stain normalization techniques were applied in order to preserve diagnostically relevant morphological features. Stain normalization was intentionally not applied in this benchmark to preserve real-world staining variability and to avoid introducing additional preprocessing assumptions.

### 3.3. Data Partitioning, Augmentation, and Class Imbalance Handling

The dataset initially comprised 569 peripheral blood smear images, including 422 Positive and 147 Negative samples. Prior to dataset partitioning, image-level integrity checks were performed to identify duplicate image groups based on content-based cryptographic hashing (MD5), where identical hash values indicate duplicate image content. These duplicates were handled prior to model training to ensure data independence.

The dataset was randomly shuffled and partitioned into training (70%), validation (15%), and test (15%) subsets using group-aware splitting implemented via scikit-learn’s GroupShuffleSplit, with grouping based on identical MD5 image hashes to preserve class proportions while preventing leakage of identical image content across subsets. The resulting splits contained 397 training images, 86 validation images, and 86 test images. Dataset partitioning was performed before any data augmentation or balancing procedures.

**Figure 4:**
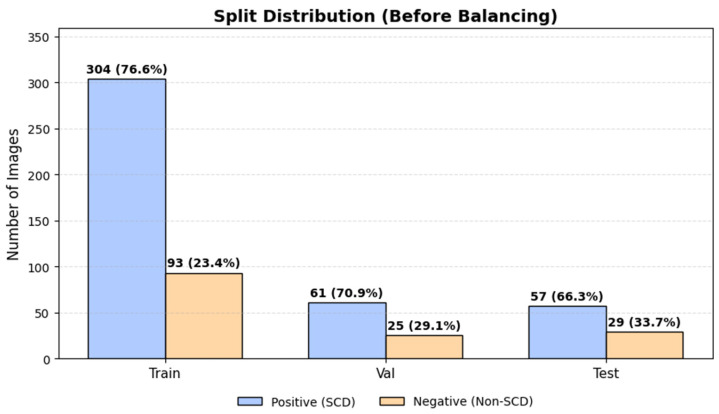
Class distribution across dataset splits prior to balancing.

Figure 4 shows the class distribution across training, validation, and test splits prior to balancing, demonstrating consistent class proportions and leakage-safe partitioning.

To prevent data leakage, strict hash-level overlap checks were conducted to ensure that no image appeared in more than one subset. Zero overlap between training, validation, and test image sets was verified programmatically, confirming the independence of all subsets. The validation and test sets were held out entirely and used exclusively for model selection and final performance evaluation, respectively.

Given the pronounced class imbalance, class balancing was applied exclusively to the training set to mitigate bias during model optimization. Specifically, the minority class (Negative) was upsampled via random resampling with replacement to match the number of Positive samples, resulting in a balanced training set of 608 images (304 per class). Importantly, no balancing, resampling, or duplication was applied to the validation or test sets, which retained their original class distributions to reflect real-world screening conditions.

Data augmentation was applied exclusively to the training set to improve model generalization while preserving the integrity of the validation and test sets. Augmentation strategies were deliberately conservative and selected to simulate realistic acquisition variability without altering diagnostically relevant erythrocyte morphology. The applied augmentations included random horizontal flipping and small-angle rotations (±10°). Augmentations that could distort cellular shape, texture, or staining characteristics were intentionally avoided. Validation and test images were subjected only to resizing and normalization to ensure unbiased performance assessment. All augmentations were applied on-the-fly during training at each epoch and were not precomputed or stored.

**Figure 5:**
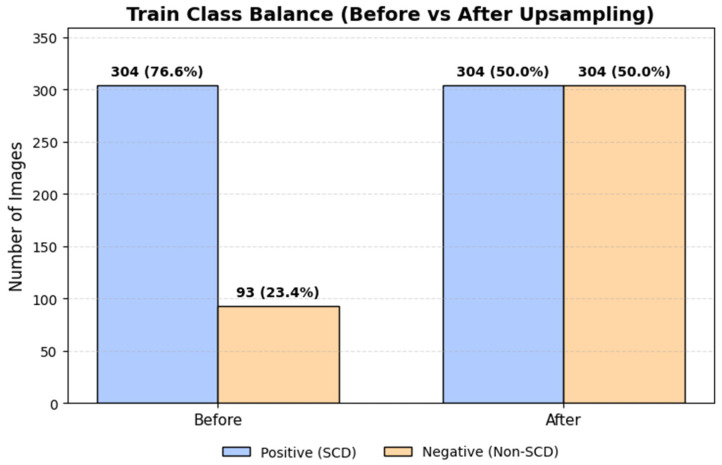
Class balance before and after training-set upsampling.

Figure 5 illustrates the class distribution in the training set before and after upsampling, confirming that class balancing was applied exclusively during training, while validation and test sets retained their original imbalanced distributions to reflect screening conditions.

Model performance was therefore evaluated using Macro-F1 score, in addition to accuracy and class-specific precision and recall, to provide a balanced assessment across minority and majority classes under clinically realistic conditions.

### 3.4. Model Architectures

Eleven pretrained deep learning architectures were selected to represent three complementary modeling paradigms: CNN-based, transformer-based, and hybrid CNN–Transformer architectures.

**Model Selection Rationale.** The selected architectures were intentionally chosen to span a wide spectrum of model capacity, computational efficiency, and inductive bias, rather than to exhaustively enumerate available models. The set includes lightweight CNNs suitable for screening deployment, medium-capacity CNNs optimized for morphological feature learning, transformer-based models emphasizing global attention, hierarchical transformers introducing localized attention, and hybrid CNN–Transformer architectures that combine local convolutional bias with global context modeling. This design supports a controlled comparison focused on robustness, stability, and screening-relevant trade-offs under data-constrained conditions, rather than peak accuracy optimization. All models use ImageNet pretraining to ensure a common initialization baseline and enable fair architectural comparison, while acknowledging potential domain shift between natural images and blood smear microscopy.

#### 3.4.1. CNN-Based Architectures

VGG16, VGG19, ResNet18, ResNet50, DenseNet121, EfficientNet-B0, MobileNetV2, and InceptionV3 were included due to their demonstrated effectiveness in medical image analysis and their convolutional inductive bias toward learning localized spatial patterns. Through spatially constrained convolutional kernels and hierarchical feature extraction, these architectures are particularly well suited for capturing fine-grained morphological characteristics of red blood cells, such as cell contour, curvature, elongation, and edge irregularities, which are central to distinguishing sickled from non-sickled erythrocytes.

#### 3.4.2. Vision Transformer (ViT)

The ViT (ViT-B/16) was selected to evaluate a purely self-attention-based architecture that models’ images as sequences of fixed-size patches and learns feature relationships through global attention mechanisms rather than spatially localized convolutions. Unlike CNNs, ViT does not impose an explicit inductive bias toward locality, translation invariance, or hierarchical feature composition. As a result, ViT relies primarily on data-driven learning to infer spatial structure and morphological patterns. Including ViT in this study enables systematic assessment of whether global context modeling alone is sufficient for discriminating subtle red blood cell morphological differences in peripheral blood smear images, particularly under limited data conditions.

#### 3.4.3. Hierarchical Transformer (Swin Transformer)

The Swin Transformer was included as a representative hierarchical ViT that introduces structured locality into self-attention through shifted window mechanisms. Unlike pure ViT, which applies global self-attention uniformly across all image patches, Swin Transformer restricts attention computation to local windows while progressively merging patches across stages, thereby forming a hierarchical feature representation. Although Swin incorporates locality-aware attention, it remains a fully transformer-based architecture and does not include convolutional layers. Its inclusion enables evaluation of whether hierarchical attention and localized self-attention alone can compensate for the absence of convolutional inductive bias in modeling red blood cell morphology.

#### 3.4.4. Hybrid CNN–Transformer Architecture (MaxViT)

MaxViT was selected as a true hybrid CNN–Transformer architecture that integrates convolutional operations with self-attention within a unified framework. Specifically, MaxViT combines convolutional MBConv blocks for efficient local feature extraction with multi-axis self-attention layers that model long-range spatial dependencies across both local windows and the full image. This design explicitly fuses convolutional inductive bias with transformer-based global context modeling. As such, MaxViT provides a principled architecture for assessing whether hybridization of local morphological feature learning and global contextual reasoning yields superior performance in red blood cell classification tasks, particularly under limited data conditions.

### 3.5. Model Training and Implementation Details

**Hardware and Software Environment.** All experiments were conducted using Python 3.13 on macOS (Apple Silicon) with PyTorch 2.8.0. Training and evaluation were performed using the Apple Metal Performance Shaders (MPS) backend. Reported computational costs reflect this hardware configuration and are intended for relative comparison across models rather than absolute benchmarking against discrete GPU platforms.

All models were initialized using ImageNet-pretrained weights to leverage prior visual knowledge and accelerate convergence. The final classification layers were replaced with task-specific fully connected layers, producing two output classes. All models were fine-tuned end-to-end, with no layers frozen during training.

Training was performed using the Adam optimizer with an initial learning rate of 1 × 10^−4^ and categorical cross-entropy loss. A step-based learning rate scheduler (StepLR) was applied with a step size of 3 epochs and a decay factor (γ = 0.1) to improve training stability and convergence consistency across architectures. Each model was trained for 10 epochs with a fixed batch size of 16.

**Hyperparameter Rationale.** A fixed training budget of 10 epochs was intentionally adopted across all architectures to enable a controlled comparison of architectural inductive biases under data-constrained conditions, rather than to achieve model-specific optimal performance. Pilot convergence inspection indicated that both training and validation losses stabilized within the first 5–8 epochs across architectures, with no systematic performance gains observed beyond 10 epochs. Extending training further primarily increased the risk of overfitting given the limited dataset size.

Early stopping was deliberately not employed, as it may introduce architecture-dependent stopping behavior and confound fair comparison across models. Instead, all architectures were trained for an identical number of epochs under the same optimization schedule to preserve experimental consistency. The learning rate and scheduler were adopted as conservative, standardized choices commonly used for fine-tuning ImageNet-pretrained models on small medical imaging datasets, rather than being individually tuned for specific architectures.

Model-specific input preprocessing was applied where required. In particular, InceptionV3 was trained at its native input resolution of 299 × 299 pixels, while all other architectures were trained with 224 × 224-pixel inputs, in accordance with their architectural specifications. Aside from input resolution, all training procedures and hyperparameters were identical across models to ensure a fair comparison. All experiments were implemented in Python using the PyTorch framework, with standard models sourced from torchvision and the MaxViT architecture implemented via the timm library. Training was conducted using GPU acceleration where available, with automatic fallback to CPU execution.

**Reproducibility.** To ensure reproducibility, fixed random seeds were used consistently across all experiments for Python, NumPy, and PyTorch (seed = 42).

All architectures were trained under an identical training budget (number of epochs, optimizer, learning rate, and scheduler) to enable controlled comparison of architectural inductive biases. This design does not aim to reflect optimal, architecture-specific tuning, particularly for transformer-based models, which may benefit from longer training or alternative optimization strategies.

The experimental pipeline was implemented in a modular and fully scripted manner to facilitate reproducibility. Code availability will be considered in future extensions of this work.

### 3.6. Evaluation Metrics and Comparative Analysis

Model performance was evaluated exclusively on a leakage-safe held-out test set that was never used during training or validation. Evaluation relied on standard classification metrics, including test accuracy, class-specific precision, recall, and F1-score for both negative and positive classes. To support balanced comparison across models, macro-averaged precision, recall, and F1-score were also reported.

Confusion matrices were generated for each model to analyze class-wise prediction behavior and error distribution, with particular emphasis on false-negative and false-positive errors relevant to sickle cell disease screening. In addition, comparative visualizations, including performance heatmaps and accuracy ranking plots, were used to highlight differences across architectures.

All architectures were trained under identical experimental conditions, including dataset splits, preprocessing pipelines, data augmentation strategies, optimization settings, and training epochs. This controlled experimental design ensured fair and reproducible comparison across convolutional, transformer-based, and hybrid architectures.

To assess robustness beyond a single split, repeated group-aware data partitions were used to rank all models based on mean performance and standard deviation. The top five models were then fixed and subjected to deeper evaluation on a seeded reference split, including bootstrap 95% confidence intervals, paired McNemar tests, ROC/PR analysis with threshold sweeps, and calibration assessment.

### 3.7. Explainable Artificial Intelligence (XAI)

To enhance model interpretability and support clinical relevance, XAI techniques were applied to the top-performing models, as measured by test-set performance. Given the architectural diversity of the evaluated models, architecture-specific explanation methods were employed to ensure meaningful and faithful interpretation of model behavior.

For CNN-based models (ResNet, DenseNet121, EfficientNet-B0, MobileNetV2, VGG16/19, and InceptionV3), Gradient-weighted Class Activation Mapping (Grad-CAM) was used to generate class-discriminative heatmaps highlighting salient blood-cell regions that most strongly influenced model predictions.

For the hybrid CNN–transformer architecture (MaxViT-Tiny), Eigen-CAM was applied to visualize the combined contribution of convolutional features and transformer-based attention, enabling interpretation of both local morphological cues and global contextual information.

For pure transformer models, architecture-appropriate attention-based explanations were employed. ViT (ViT-B/16) predictions were interpreted using attention rollout, which reveals patch-level global importance across the input image. For the Swin Transformer, Grad-CAM was computed from the output of the final Swin stage in the TorchVision implementation (target layer: model.features[−1]), with an appropriate token-to-spatial reshape transform applied to generate region-level importance maps within the shifted-window attention framework.

XAI visualizations were generated primarily for misclassified test samples from both classes to analyze error patterns and decision behavior under challenging cases. These explanations suggest that different architectural paradigms tend to emphasize distinct spatial regions and feature cues, enabling a qualitative comparison of model focus across CNN-based, hybrid, and transformer-based architectures.

## 4. Results

This section presents a comprehensive evaluation of CNN, transformer-based, and hybrid CNN–Transformer architectures for image-level sickle cell disease classification. The evaluation follows a staged, leakage-safe protocol designed to assess baseline performance, robustness under data variability, and detailed model behavior.

Specifically, all models were benchmarked under a leakage-safe baseline split to establish an initial performance reference. Robustness was then assessed using repeated group-aware splits, with mean and standard deviation (mean ± SD) reported to quantify stability across runs. Based on this global robustness analysis, a subset of top-performing models was fixed using mean accuracy as the primary criterion and macro-F1 score as a secondary tie-breaker.

Subsequent analyses focused on this Top-5 subset and included class-wise performance metrics, detailed error analysis, and deeper statistical evaluation using confidence intervals, paired McNemar tests, ROC and precision–recall analysis, and calibration assessment. This multi-stage evaluation framework ensures that conclusions are based on consistent performance trends rather than single-split outcomes.

### 4.1. Robustness Analysis Using Repeated Group-Aware Splits

To reduce reliance on a single train–test split and to assess model stability under data variability, all evaluated architectures were evaluated using repeated, leakage-safe, group-aware data splits. For each model, test accuracy and class-wise performance metrics were computed across multiple splits, and the mean and standard deviation (mean ± SD) were reported.

Table 3 summarizes the mean performance across repeated splits for all eleven evaluated models, including test accuracy and macro-averaged precision, recall, and F1-score. Reporting mean ± SD provides a more robust estimate of expected performance and highlights the relative stability of each architecture under limited data conditions.

Overall, convolutional and hybrid architectures demonstrated more stable performance with lower variance across splits compared to pure transformer-based models. The hybrid MaxViT-Tiny achieved the highest mean test accuracy (0.935 ± 0.026), followed closely by DenseNet121 (0.933 ± 0.021), InceptionV3 (0.933 ± 0.022), and MobileNetV2 (0.933 ± 0.021). While absolute performance differences among the top models were modest, the repeated-split evaluation revealed consistent trends favoring convolutional inductive bias and hybrid designs for morphology-driven blood smear classification tasks.

Based on this global robustness analysis, the top five models were selected according to mean test accuracy across repeated splits, with macro-F1 used as a complementary stability indicator, and were subsequently carried forward for detailed statistical evaluation, error analysis, and explainability assessment.

**Figure 6:**
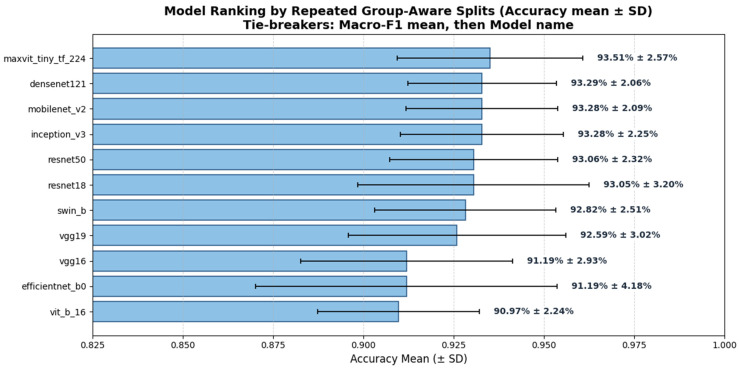
Model ranking by mean test accuracy (±SD) across repeated group-aware splits.

Figure 6 visualizes the global ranking of all evaluated models based on mean test accuracy across repeated group-aware splits, including variability estimates (±SD), and illustrates the relative stability of convolutional, hybrid, and transformer-based architectures.

### 4.2. Single-Split Benchmark Results (Reference Evaluation)

In addition to the robustness analysis based on repeated group-aware splits, a reference evaluation was conducted using a single, leakage-safe held-out test split (*n* = 86). This evaluation provides a fixed comparison point for detailed error analysis, statistical testing, and explainability assessment in subsequent sections.

Table 4 reports the performance of all eleven evaluated architectures on this reference test set in terms of test accuracy and macro-averaged precision, recall, and F1-score. Overall, convolutional and hybrid models again demonstrated superior performance compared to pure transformer-based architectures.

MaxViT-Tiny achieved the highest test accuracy (0.942), followed by InceptionV3 and ResNet18 (both 0.930). DenseNet121, while not the top-ranked model under this single split, maintained strong balanced performance across classes, supporting its consistent behavior observed in the repeated-split robustness analysis.

In contrast, the pure ViT (ViT-B/16) exhibited substantially lower performance under this fixed split, further highlighting the sensitivity of transformer-only architectures to limited training data in morphology-driven blood smear classification tasks.

**Figure 7:**
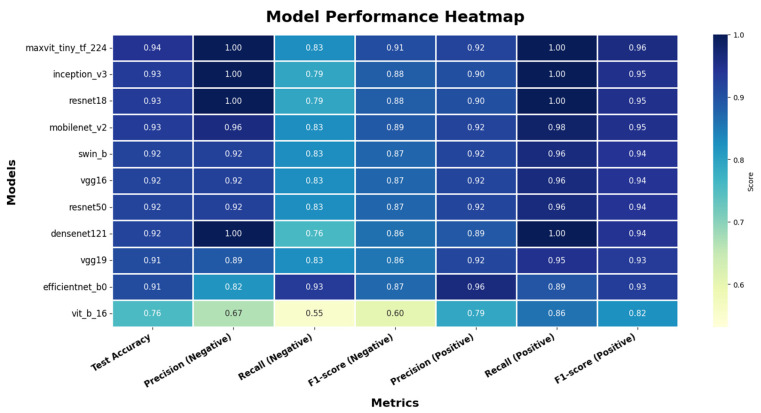
Model performance heatmap.

Figure 7 presents a comprehensive model performance heatmap summarizing test accuracy and class-wise precision, recall, and F1-scores for all evaluated architectures on the reference test split. The visualization highlights strong, balanced performance for convolutional and hybrid models, while pure transformer-based models exhibit comparatively lower, less stable performance.

**Figure 8:**
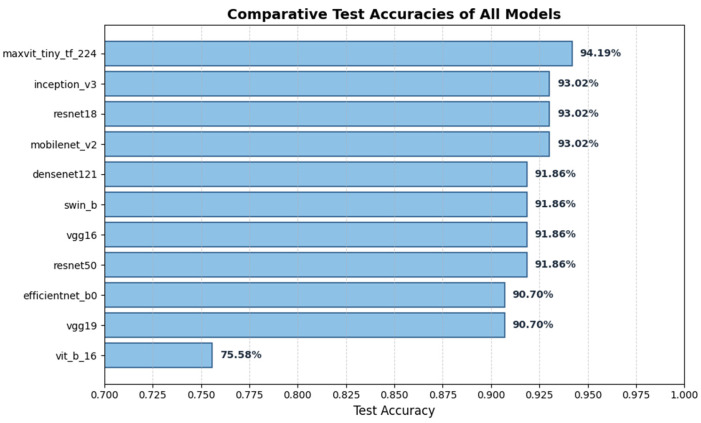
Test-accuracy bar chart.

Figure 8 illustrates comparative test accuracies across models, confirming the relative ranking observed in both the single-split and repeated-split evaluations.

However, based on the robustness analysis across repeated splits, the top five models (MaxViT-Tiny, DenseNet121, InceptionV3, MobileNetV2, and ResNet50) were selected for detailed statistical testing, error analysis, and explainability evaluation in the following sections, as ranked in Figure 6.

### 4.3. Top-5 Model Selection Based on Repeated-Split Ranking

Based on the repeated, leakage-safe, group-aware split analysis, a subset of five models was fixed for deeper evaluation. Model selection was driven primarily by mean test accuracy across repeated splits, with macro-F1 score applied as a secondary tie-breaker to favor balanced class-wise performance.

It is important to note that the performance differences among the top-ranked models are relatively small, and their rankings may vary slightly across different runs due to the inherent variability introduced by repeated data splits. Such variations are expected in data-constrained settings and reflect statistical fluctuation rather than instability in the experimental design or implementation.

Accordingly, the Top-5 models reported in this study correspond to the results obtained from the current execution of the repeated-split evaluation pipeline, conducted under fixed seeds and identical experimental settings. The overall conclusions are therefore derived from consistent performance trends observed across repeated splits, rather than reliance on a single deterministic ranking.

The resulting Top-5 models were:

- MaxViT-Tiny—93.51% ± 2.57%
- DenseNet121—93.29% ± 2.06%
- InceptionV3—93.28% ± 2.25%
- MobileNetV2—93.28% ± 2.09%
- ResNet50—93.06% ± 2.32%

These models consistently ranked highest in the global robustness analysis and demonstrated both strong performance and relative stability under data variability. Focusing subsequent analyses on this subset enables more rigorous statistical evaluation while avoiding overinterpretation of minor differences across the full model pool.

Accordingly, the selected Top-5 models were subjected to deeper evaluation using a fixed, seeded reference test split, including statistical uncertainty estimation, paired significance testing, detailed error analysis, and explainability assessment. This design allows controlled, interpretable comparisons while maintaining strict data leakage prevention and consistency across evaluation stages.

### 4.4. Learning Dynamics and Confusion Matrix Analysis (Top-5 Models)

Learning curves and confusion matrices were analyzed for the Top-5 models selected based on the repeated group-aware split ranking to further examine training stability, convergence behavior, and class-wise prediction patterns under a fixed reference split.

**Figure 9:**
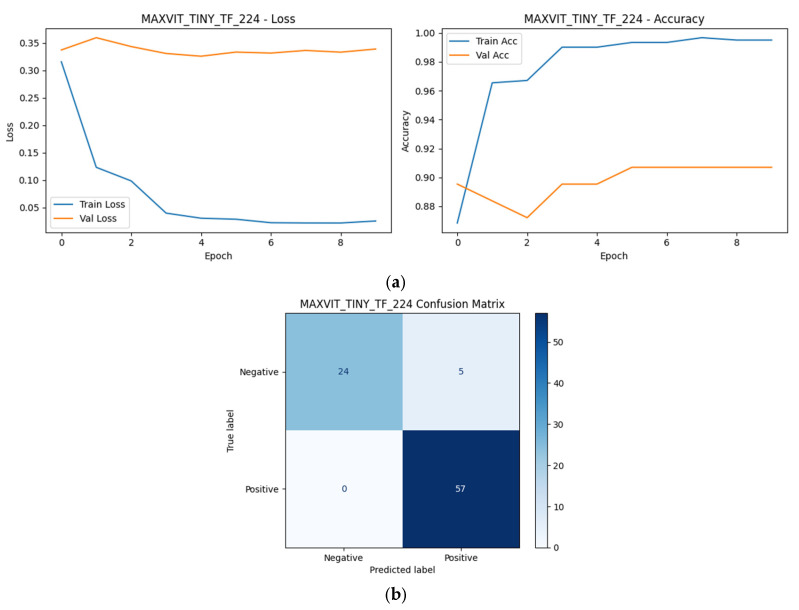
Training dynamics and confusion matrix for MaxViT-Tiny on the reference test split. (**a**) Learning curves showing training and validation loss and accuracy across epochs. (**b**) Confusion matrix illustrating class-wise prediction performance.

**Figure 10:**
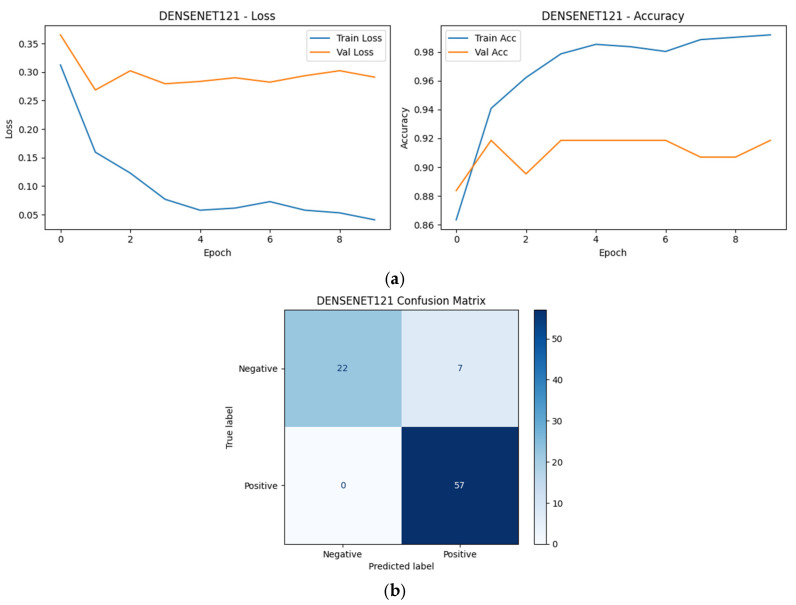
Training dynamics and confusion matrix for DenseNet121 on the reference test split. (**a**) Learning curves showing stable convergence behavior. (**b**) Confusion matrix highlighting strong sensitivity for the positive class.

**Figure 11:**
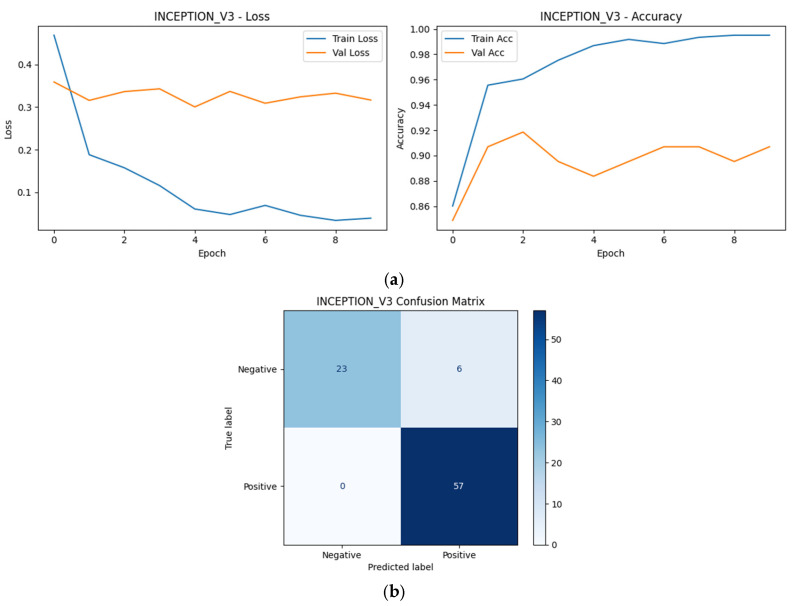
Training dynamics and confusion matrix for InceptionV3 on the reference test split. (**a**) Learning curves showing stable convergence behavior. (**b**) Confusion matrix highlighting strong sensitivity for the positive class.

**Figure 12:**
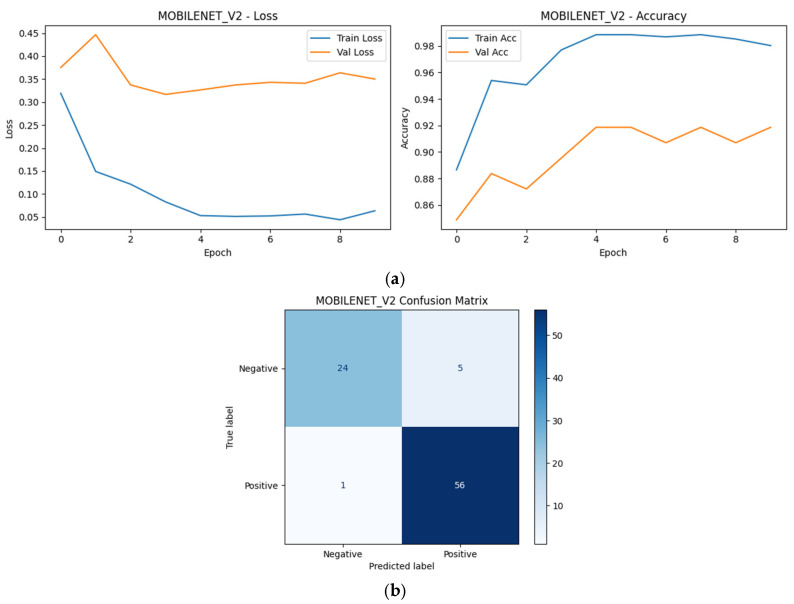
Training dynamics and confusion matrix for MobileNetV2 on the reference test split. (**a**) Learning curves indicating rapid convergence and stable validation accuracy. (**b**) Confusion matrix reflecting competitive performance despite the lightweight architecture.

**Figure 13:**
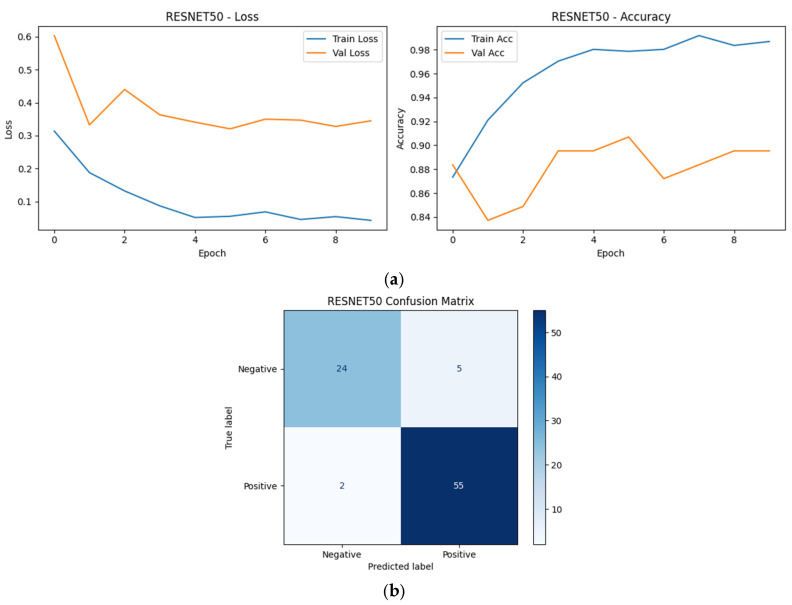
Training dynamics and confusion matrix for ResNet50 on the reference test split. (**a**) Learning curves demonstrating consistent optimization behavior. (**b**) Confusion matrix showing class-wise prediction outcomes.

As shown in Figure 9, Figure 10, Figure 11, Figure 12 and Figure 13, all selected models exhibit stable optimization behavior, characterized by a monotonic decrease in training loss and convergence of validation accuracy within the allocated training epochs. No clear signs of severe overfitting were observed, indicating that the adopted training protocol and regularization strategies were adequate for the dataset size.

The corresponding confusion matrices reveal consistently high sensitivity for the positive (SCD) class across all Top-5 models, which is a critical requirement for screening-oriented applications. Most observed errors correspond to Negative → Positive misclassifications, reflecting a conservative prediction tendency that prioritizes sensitivity over specificity. Such behavior is desirable in initial screening scenarios, where false positives can be resolved through confirmatory testing.

Although the overall performance of the Top-5 models is closely clustered, subtle differences in error distribution and convergence dynamics can be observed, reflecting architectural differences in feature learning and inductive bias. These observations complement the quantitative robustness and single-split results presented earlier and motivate the deeper statistical and explainability analyses presented in subsequent sections.

### 4.5. Error Analysis of Top-5 Models

A structured error analysis was conducted for the Top-5 models selected based on the repeated group-aware split ranking (Section 4.3), using the fixed, leakage-safe reference test split (*n* = 86). The analysis aimed to systematically characterize failure modes by examining misclassification types, prediction confidence in erroneous cases, and cross-model overlap of misclassified samples under screening-oriented conditions.

#### 4.5.1. Error Type Distribution

**Figure 14:**
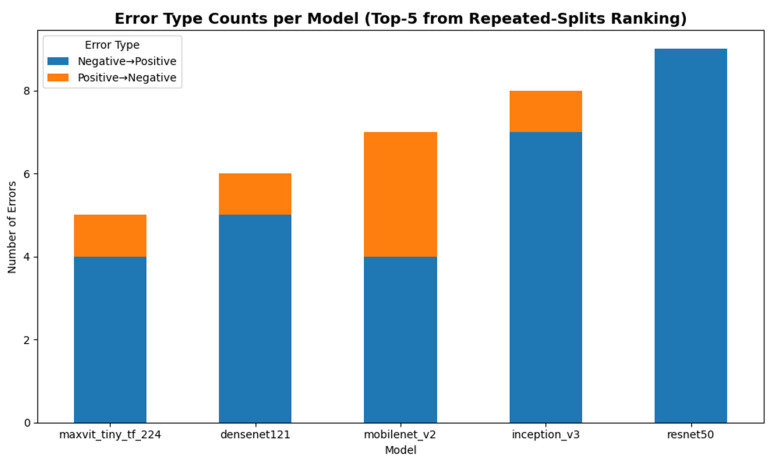
Error type counts (Negative → Positive vs. Positive → Negative) for the Top-5 models on the reference test split.

Figure 14 summarizes the distribution of misclassification types for the Top-5 models. Across all evaluated architectures, Negative → Positive errors (false positives) were consistently more frequent than Positive → Negative errors (false negatives). This pattern reflects a conservative classification tendency favoring sensitivity over specificity, which is clinically desirable in screening scenarios where failing to detect true SCD cases is more consequential than generating additional follow-up investigations.

Among the Top-5 models, MaxViT-Tiny exhibited the lowest number of misclassifications (5/86), followed by DenseNet121 (6/86), MobileNetV2 (7/86), InceptionV3 (8/86), and ResNet50 (9/86). Despite these small differences in absolute error counts, all models demonstrated a similar error structure dominated by false positives, indicating consistent screening-oriented behavior across architectural families.

#### 4.5.2. Qualitative Categorization of Misclassified Samples

**Figure 15:**
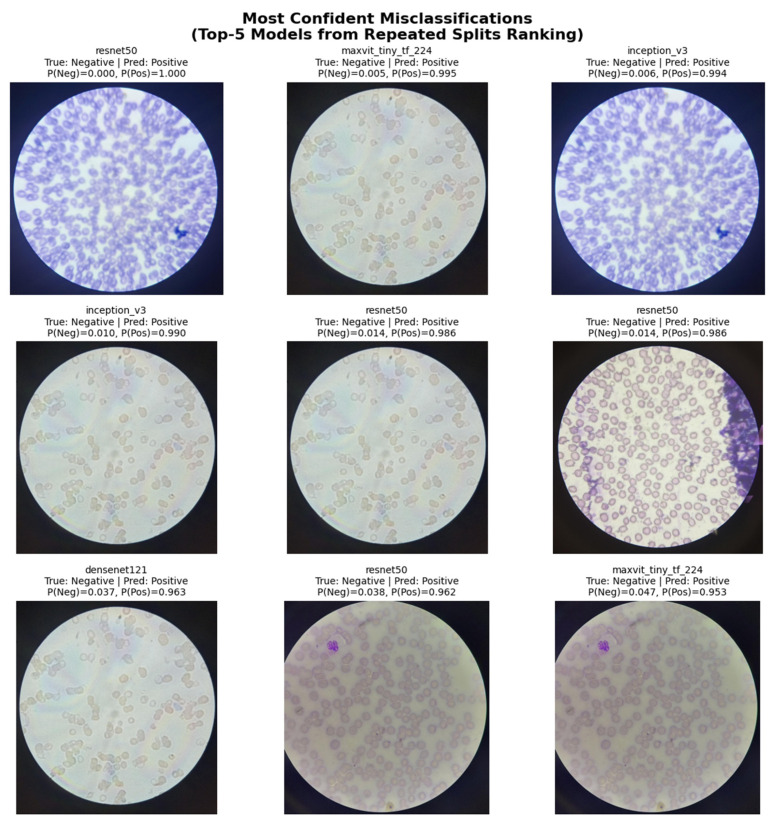
Examples of the most confident misclassifications for the Top-5 models. Each image is annotated with the true label, predicted label, and predicted class probabilities.

Qualitative inspection of high-confidence misclassified samples (i.e., predictions with strong class probability despite incorrect labels; Figure 15) revealed recurring visual characteristics that contributed to erroneous predictions across models. These error cases frequently involved (i) overlapping or clustered red blood cells, which obscure individual cell boundaries; (ii) staining artifacts or uneven illumination that distort color and texture cues; and (iii) borderline morphological variations where normal erythrocytes partially resemble sickled shapes. Such visual ambiguities can plausibly confound automated classification models operating at the image level.

These qualitative categorizations were based on visual inspection, not independently validated by hematology experts, and are therefore intended to be exploratory rather than definitive.

Although no explicit quantitative image-quality metrics were computed, misclassified samples were qualitatively associated with common image-quality proxies, including uneven staining intensity, illumination variability, and cell overlap. These factors are inherent to real-world PBS preparation and likely contribute to ambiguity at the image level.

#### 4.5.3. Prediction Confidence in Misclassifications

Analysis of predicted class probabilities for misclassified samples showed that several errors were associated with high model confidence, particularly in false-positive cases where negative images received strong positive-class probabilities. This observation suggests that certain non-sickled morphological patterns systematically trigger features learned as indicative of sickling, underscoring the challenge of distinguishing subtle morphological variations under heterogeneous imaging conditions.

#### 4.5.4. Cross-Model Error Overlap

Cross-model error overlap analysis, performed using image-hash–based matching, revealed that a subset of test images was consistently misclassified by multiple architectures. The largest overlap was observed between InceptionV3 and ResNet50, followed by substantial overlap involving DenseNet121 and MobileNetV2. The recurrence of identical misclassified samples across diverse architectures suggests that these errors are driven primarily by intrinsic dataset-level challenges rather than model-specific weaknesses or unstable training behavior.

#### 4.5.5. Summary

Overall, the error analysis indicates that the Top-5 models share highly consistent failure patterns characterized by a bias toward false-positive errors and sensitivity-preserving behavior. The convergence of error types and overlapping misclassified samples across architectures supports the robustness of the experimental design and suggests that further performance gains are likely to depend on improved handling of ambiguous morphological cases, rather than architectural changes alone.

### 4.6. Statistical Uncertainty and Screening-Oriented Evaluation (Top-5 Models)

To quantify statistical uncertainty and screening relevance, an extended evaluation was conducted for the Top-5 models using the fixed, leakage-safe reference test split (*n* = 86). This analysis complements point estimates by characterizing uncertainty bounds, class-wise trade-offs, and probability calibration.

Non-parametric bootstrap resampling (1000 iterations) was used to estimate 95% confidence intervals (CI) for accuracy, macro-F1 score, sensitivity, specificity, and false-positive rate. As summarized in Table 5, the confidence intervals of the Top-5 models show substantial overlap, indicating that observed performance differences are modest and should be interpreted cautiously given the limited test size.

**Figure 16:**
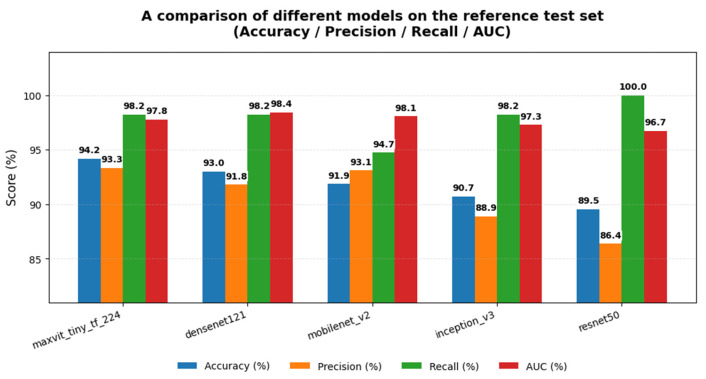
Comparison of accuracy, precision, recall, and AUC for the Top-5 models on the reference test set.

Figure 16 provides a visual comparison of accuracy, precision, recall, and AUC for the Top-5 models on the reference test split, complementing the tabulated results.

Paired exact McNemar tests were performed for all Top-5 model comparisons on the reference split. No statistically significant differences were observed after Bonferroni correction (Table 6), confirming that the models exhibit comparable per-sample performance despite small numerical differences.

**Figure 17:**
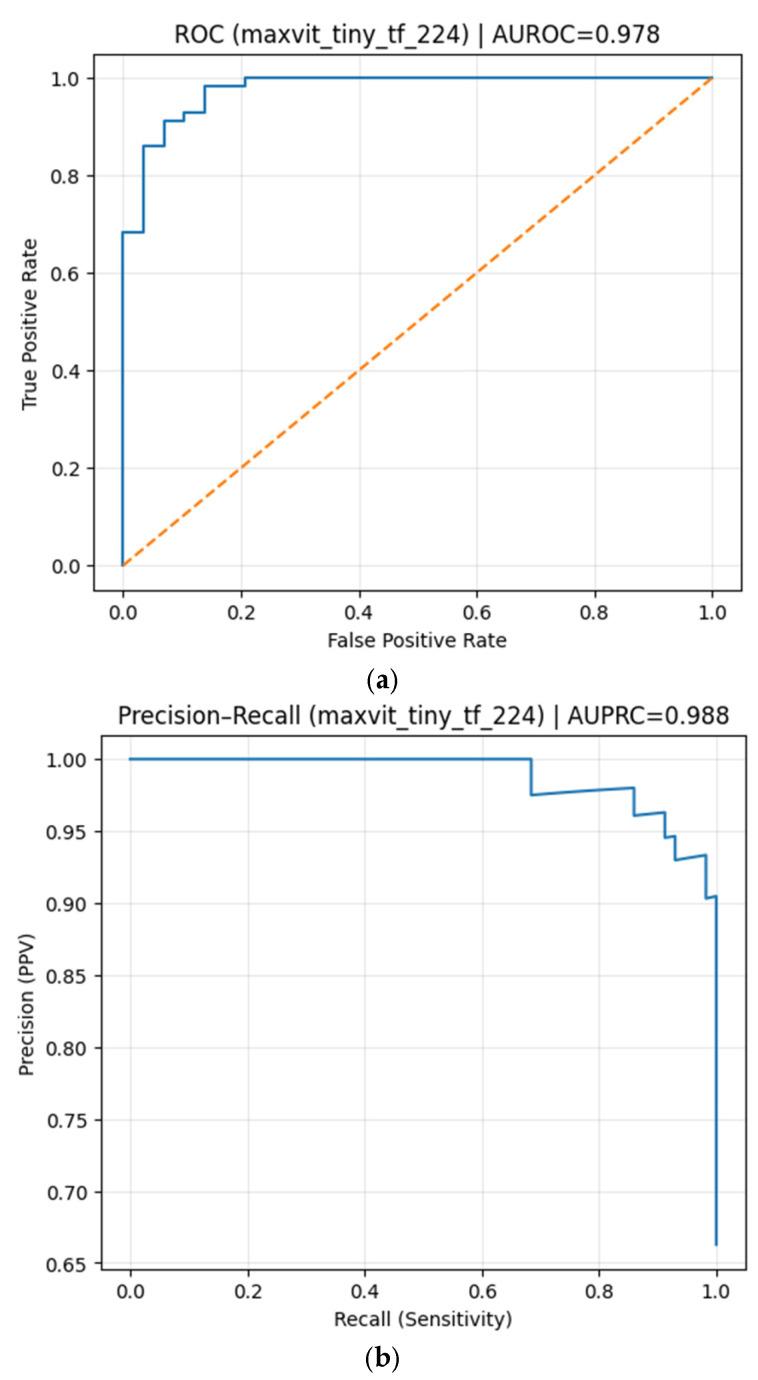
Screening-oriented discrimination performance for MaxViT-Tiny on the reference test split: (**a**) Receiver operating characteristic (ROC) curve (AUROC = 0.978) and (**b**) Precision–recall curve (AUPRC = 0.988). The dashed diagonal line in (**a**) represents the performance of a random classifier.

**Figure 18:**
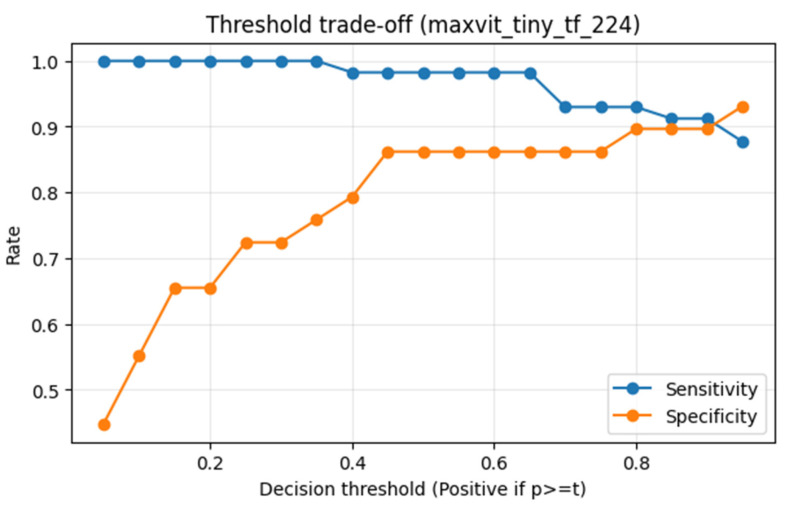
Threshold Sensitivity–Specificity Trade-off for MaxViT-Tiny.

Screening-oriented discrimination was further assessed for the best-ranked model under repeated-split analysis (MaxViT-Tiny). As shown in Figure 17, the model achieved high AUROC and AUPRC values, indicating strong class separability under class imbalance. As shown in Figure 17, MaxViT-Tiny achieved strong screening-oriented discrimination with AUROC = 0.978 and AUPRC = 0.988 on the reference test split, indicating high separability under class imbalance. A threshold sweep analysis (Figure 18) demonstrated clear sensitivity–specificity trade-offs, supporting threshold selection based on screening priorities rather than a fixed operating point.

These results support a confidence-aware triage strategy in which high-confidence positives prompt confirmatory testing, while uncertain cases are flagged for manual review, allowing sensitivity–specificity trade-offs to be adjusted according to screening priorities.

In addition to predictive performance, computational efficiency is an important consideration for screening-oriented deployment. Table 7 summarizes the training cost and approximate model complexity of the Top-5 architectures under identical experimental settings.

**Figure 19:**
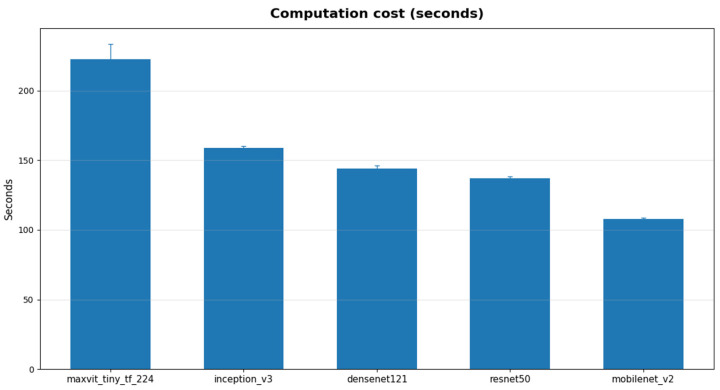
Training time and computational cost per epoch for the Top-5 models.

Figure 19 summarizes the computational cost of training the Top-5 models, highlighting trade-offs between predictive performance and computational efficiency relevant for screening deployment.

**Figure 20:**
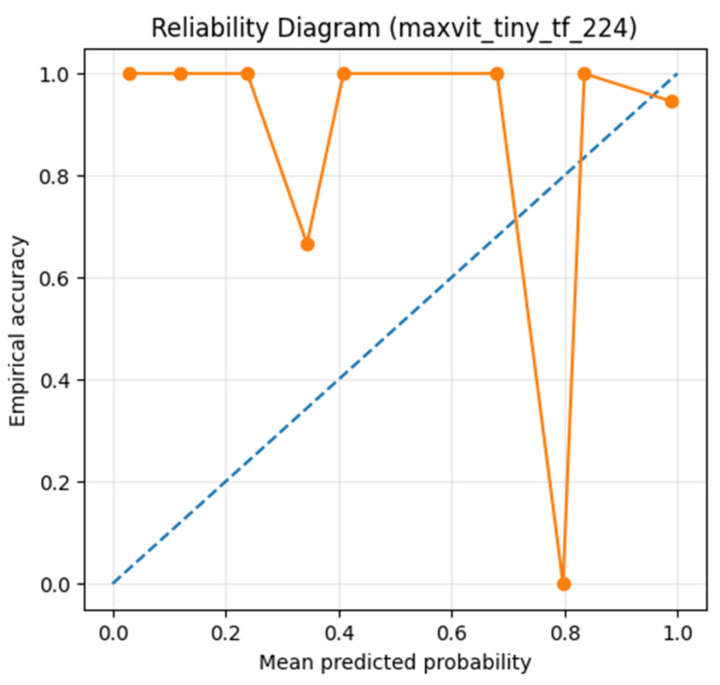
Calibration analysis for MaxViT-Tiny using a reliability diagram. The dashed diagonal line indicates perfect calibration, where predicted probabilities match empirical accuracy. Mild deviations from this line suggest slight overconfidence in certain probability ranges.

Finally, calibration analysis using the Brier score, Expected Calibration Error (ECE), and reliability diagrams (Figure 20) indicated generally good probabilistic behavior, with mild overconfidence in certain probability ranges. This observation aligns with the dominance of false-positive errors and motivates confidence-aware or triage-based screening strategies.

Similar calibration trends were observed across other top-performing models, and MaxViT-Tiny is shown here as a representative example.

Overall, these results indicate that the Top-5 models exhibit statistically comparable performance within uncertainty bounds, and that thresholding and calibration are critical considerations for screening-oriented deployment.

### 4.7. Explainability Analysis (Top-5 Models)

The explainability analysis was explicitly designed to address the research question of whether convolutional neural networks focus more tightly on RBC morphology compared with transformer-based architectures.

To provide qualitative insight into model decision behavior and to complement the quantitative evaluation, explainable artificial intelligence (XAI) techniques were applied to the Top-5 models selected from the repeated-split robustness analysis. Given the architectural diversity of the selected models, architecture-appropriate explanation methods were employed to ensure faithful and interpretable visualization of model attention.

For CNN-based architectures, Gradient-weighted Class Activation Mapping (Grad-CAM) was used to generate class-discriminative heatmaps indicating spatial regions that contributed most strongly to model predictions. For the hybrid CNN–Transformer architecture (MaxViT-Tiny), Eigen-CAM was applied to capture the combined influence of convolutional feature extraction and transformer-based attention mechanisms. Transformer-only architectures were analyzed using attention-based visualization techniques; however, detailed qualitative comparisons in this section focus on the Top-5 models retained for deep evaluation.

XAI visualizations were generated for representative correctly classified and misclassified samples from the reference test split. The results are interpreted qualitatively and descriptively, without assuming causal validation, and are intended to illustrate general patterns of feature utilization rather than definitive localization accuracy.

Overall, CNN-based models tend to focus on compact, localized red blood cell regions, which appears consistent with their strong inductive bias toward morphology-driven feature learning. In contrast, the hybrid MaxViT-Tiny model appears to distribute attention across both local cellular structures and broader contextual regions, reflecting its integrated convolutional and attention-based design. These observations are qualitative in nature and should be interpreted as suggestive rather than confirmatory.

#### Explainable AI Visualizations for Top-5 Models

**Figure 21:**
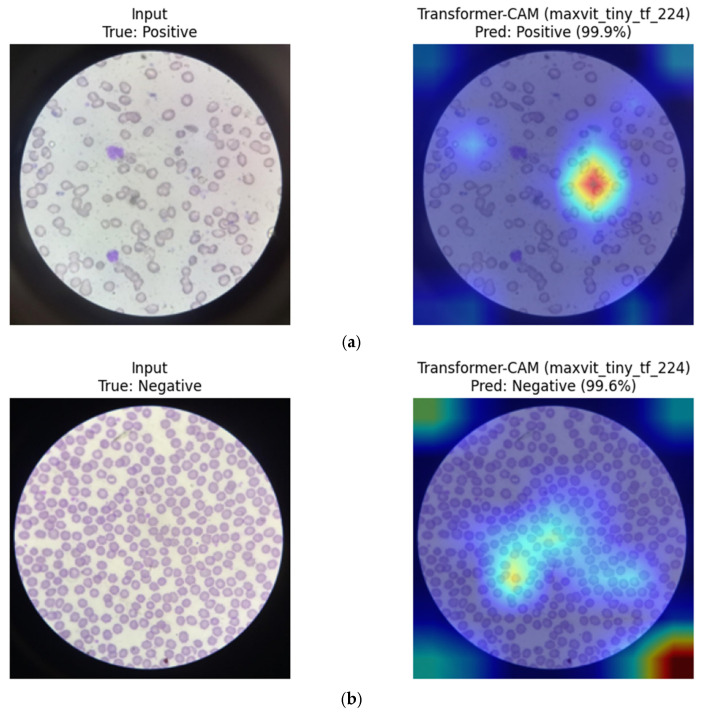
Eigen-CAM visualizations for MaxViT-Tiny. (**a**) Positive sample, (**b**) Negative sample. Warmer colors (e.g., red/yellow) indicate regions with higher contribution to the model prediction, while cooler colors (e.g., blue) represent lower relevance.

**Figure 22:**
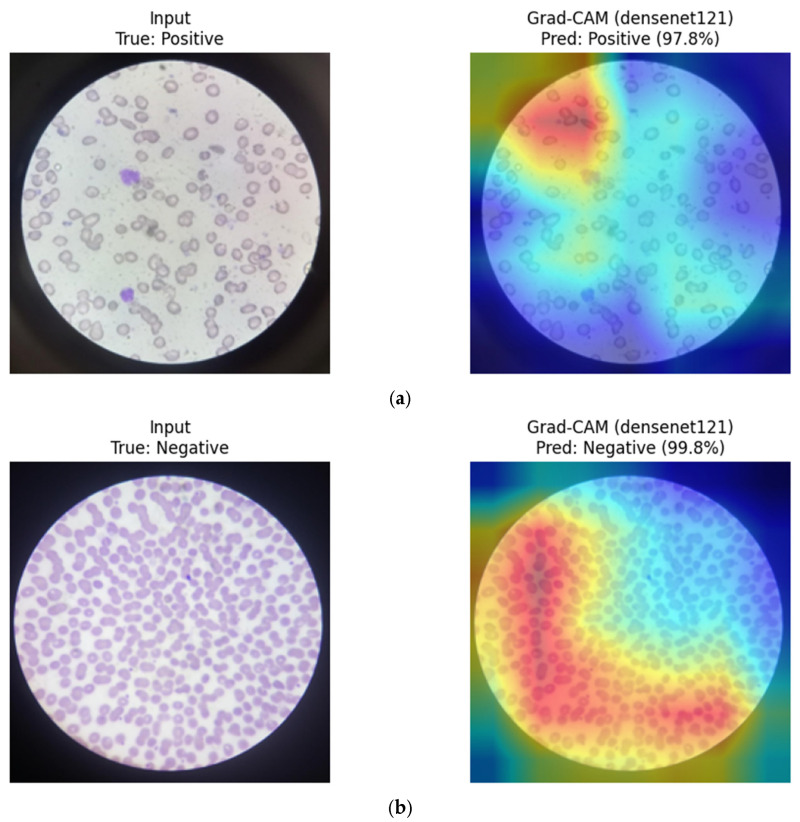
Grad-CAM visualizations for DenseNet121. (**a**) Positive sample, (**b**) Negative sample.

**Figure 23:**
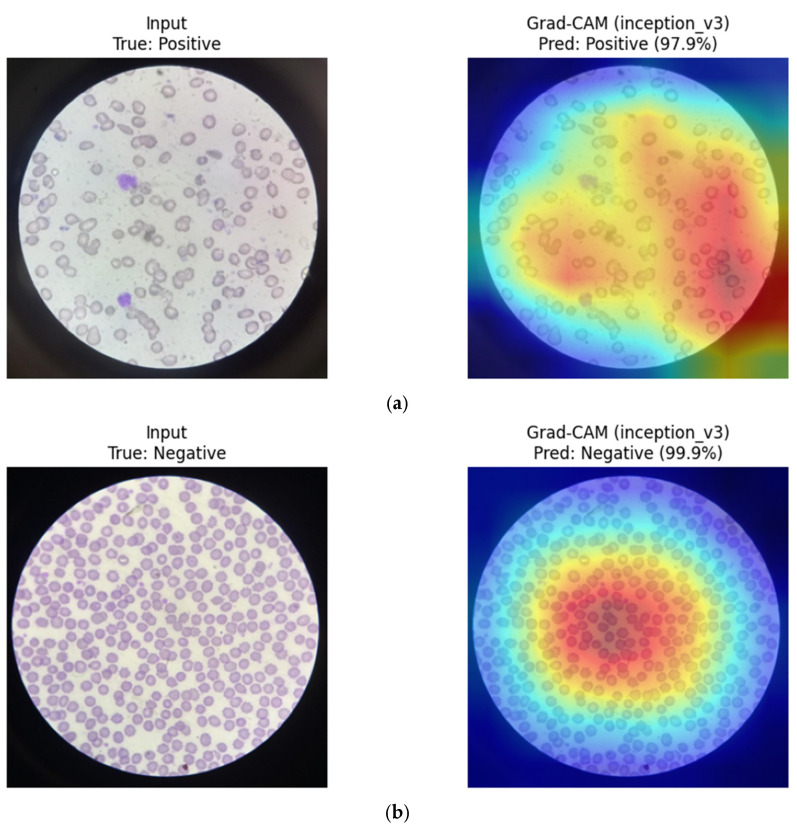
Grad-CAM visualizations for InceptionV3. (**a**) Positive sample, (**b**) Negative sample.

**Figure 24:**
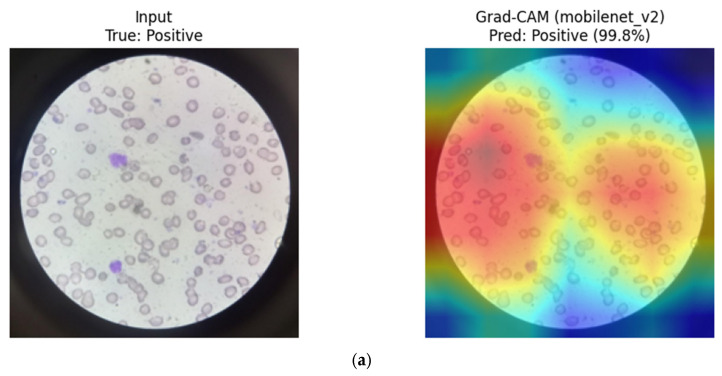

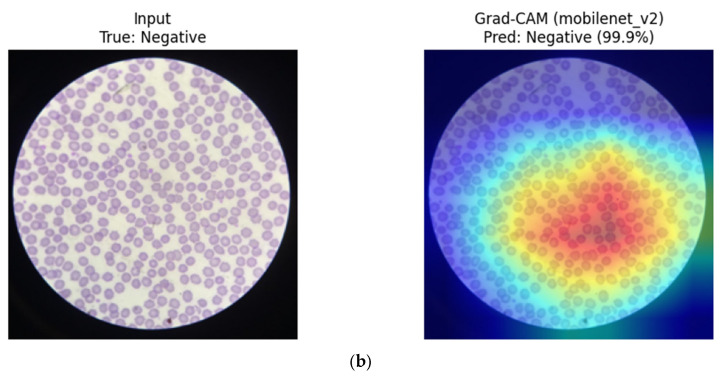
Grad-CAM visualizations for MobileNetV2. (**a**) Positive sample, (**b**) Negative sample.

**Figure 25:**
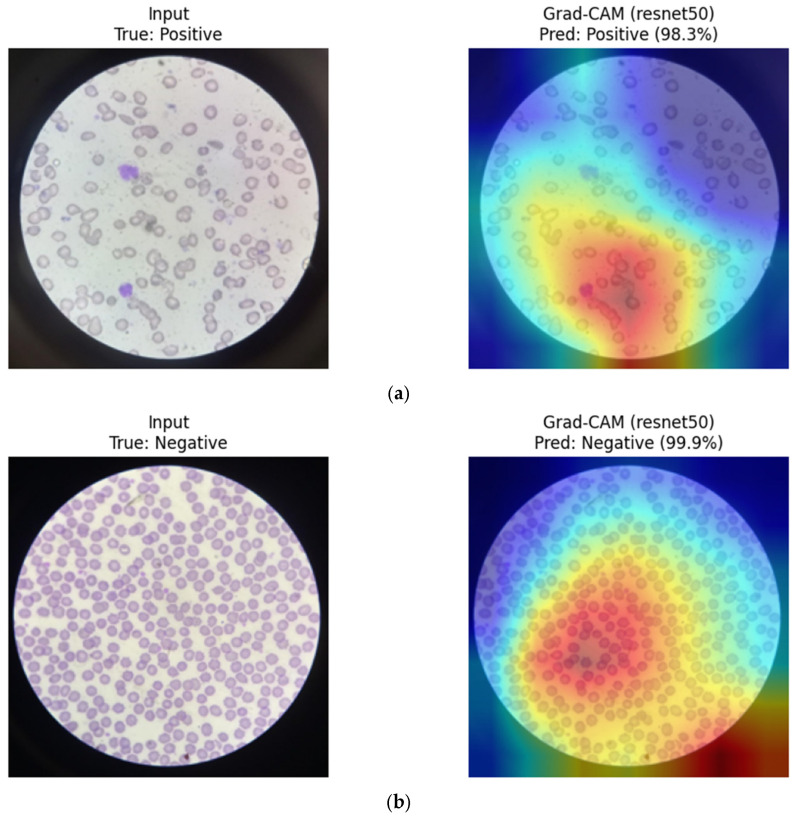
Grad-CAM visualizations for ResNet50. (**a**) Positive sample, (**b**) Negative sample.

- MaxViT-Tiny (Hybrid CNN–Transformer): Eigen-CAM visualizations for MaxViT-Tiny indicate a combination of localized focus on individual erythrocytes and broader contextual awareness across the blood smear. The model appears to integrate fine-grained morphological cues with surrounding spatial context, which may contribute to its stable performance across repeated group-aware splits and balanced sensitivity–specificity trade-off (Figure 21).

- DenseNet121 (CNN): Grad-CAM visualizations for DenseNet121 suggest that the model emphasizes localized red blood cell regions exhibiting morphological characteristics associated with sickling. Activation maps appear concentrated around elongated or irregular cell contours in positive samples, while more rounded and homogeneous erythrocytes are highlighted in negative cases. This focused attention pattern is consistent with DenseNet’s dense connectivity and strong convolutional inductive bias toward morphology-driven feature learning (Figure 22).

- InceptionV3 (CNN): Grad-CAM results for InceptionV3 show multi-scale attention patterns, with activation distributed across both individual erythrocytes and neighboring cellular structures. This behavior reflects the model’s use of parallel convolutional filters operating at different receptive field sizes, enabling capture of morphological features at varying spatial scales. Such patterns may explain its competitive performance under the repeated-split evaluation despite limited training data (Figure 23).

- MobileNetV2 (CNN): Grad-CAM visualizations for MobileNetV2 reveal compact and efficient activation regions, typically centered on a small number of discriminative erythrocytes. The attention maps appear less diffuse compared to deeper CNNs, consistent with the model’s lightweight architecture and depthwise separable convolutions. Despite its reduced capacity, MobileNetV2 appears to capture key morphological cues relevant for SCD classification (Figure 24).

- ResNet50 (CNN): Grad-CAM visualizations for ResNet50 indicate broader spatial activation across multiple erythrocytes within the field of view. While the model highlights relevant sickling patterns, attention is often distributed across several cells rather than tightly localized. This behavior may contribute to its slightly higher false-positive tendency observed in the error analysis, particularly in images with overlapping or densely packed cells (Figure 25).

To facilitate a structured qualitative comparison, identical representative test samples were visualized across CNN-based, transformer-based, and hybrid architectures using consistent XAI settings. For the same input images, CNN-based models (e.g., DenseNet121, ResNet) consistently exhibited highly localized activation patterns centered on individual red blood cells and their morphological contours. In contrast, transformer-based models (ViT, Swin) showed more spatially diffuse attention, often extending beyond individual cells to include surrounding background regions and contextual structures. Hybrid architectures such as MaxViT-Tiny demonstrated intermediate behavior, combining localized cellular focus with broader contextual awareness. While qualitative in nature, these consistent patterns across identical samples suggest that convolutional inductive bias promotes tighter morphological attention in RBC-focused tasks, whereas attention-based models distribute relevance more globally.

Overall, these qualitative XAI suggest consistent architectural trends rather than definitive localization validation. Similar activation patterns observed across multiple models for certain misclassified samples further indicate that some errors may stem from intrinsic image ambiguity rather than model-specific shortcomings. However, these observations are descriptive rather than confirmatory and should be interpreted as exploratory in the absence of quantitative localization validation.

### 4.8. Summary of Findings

This study benchmarked CNN, transformer, and hybrid architectures for sickle cell disease classification using leakage-safe evaluation and repeated group-aware splits. Results consistently showed that CNN and hybrid models outperform pure transformers under limited data conditions. Performance differences among the top models were small and largely overlapping, indicating comparable robustness rather than a single dominant architecture.

Based on repeated-split stability, MaxViT-Tiny, DenseNet121, InceptionV3, MobileNetV2, and ResNet50 were selected for deeper analysis. These models achieved high sensitivity for SCD detection, with most errors arising from visually ambiguous samples rather than model-specific failures. Statistical testing confirmed no significant differences among the Top-5, highlighting the importance of robustness, uncertainty, and threshold selection for screening use. Overall, the findings support CNN and hybrid architectures as reliable candidates for Top-3 Models.

DenseNet121 (CNN): Grad-CAM highlights localized discriminative redAI-assisted blood smear screening, while emphasizing cautious interpretation of small performance gaps.

## 5. Discussion

This study presents a controlled and leakage-safe benchmark of convolutional, transformer-based, and hybrid CNN–Transformer architectures for SCD classification from peripheral blood smear images. By enforcing identical preprocessing, training budgets, and evaluation protocols, the analysis was intentionally designed to isolate architectural effects under data-constrained conditions typical of hematological imaging tasks.

Across repeated group-aware splits, convolutional and hybrid architectures consistently demonstrated more stable performance than pure transformer models. DenseNet121 and MaxViT-Tiny ranked among the most robust models, exhibiting high mean accuracy and Macro-F1 scores with comparatively lower variance across splits. These findings highlight the importance of convolutional inductive bias—particularly localized receptive fields and hierarchical feature extraction—for morphology-driven tasks in which diagnostically relevant information is encoded in subtle variations in RBC shape.

Pure transformer models (ViT-B/16) showed reduced performance and greater sensitivity to data variability, consistent with prior observations that transformers typically require larger datasets or domain-specific pretraining to effectively model spatial structure. Hierarchical transformers (Swin-B) partially mitigated this limitation through localized attention windows, yet still did not consistently outperform CNN-based or hybrid approaches in this setting. Collectively, these results indicate that attention mechanisms alone may be insufficient for fine-grained blood smear analysis when training data are limited.

Importantly, the repeated-split evaluation revealed that performance differences among the top-ranked models were modest and statistically overlapping. Bootstrap confidence intervals and paired McNemar tests did not identify statistically significant differences between the Top-5 models after correction for multiple comparisons. Minor changes in model ranking across repeated runs are therefore expected and reflect statistical variability rather than instability in the experimental design or implementation. Accordingly, conclusions are drawn from consistent performance trends rather than reliance on a single deterministic ranking.

Although MaxViT-Tiny achieved the highest overall performance, DenseNet121 consistently demonstrated strong and stable results across repeated splits. This behavior is likely attributable to its dense connectivity pattern, which encourages feature reuse and improves gradient propagation, enabling efficient learning of subtle RBC morphological features under limited data conditions. Compared with deeper residual architectures, DenseNet121 provides competitive representational capacity with a moderate number of parameters, reducing overfitting risk while maintaining robustness.

From a computational perspective, the evaluated architectures span distinct complexity regimes. DenseNet121 falls within a medium-size class (≈8 million parameters), substantially smaller than deeper residual networks such as ResNet50 (≈25 million parameters) and hybrid architectures like MaxViT-Tiny, while remaining more expressive than lightweight models such as MobileNetV2. Although FLOPs were not explicitly optimized in this benchmark, transformer-based and hybrid architectures are expected to incur higher theoretical computational cost due to attention mechanisms, which is consistent with the observed runtime behavior. Empirical runtime measurements indicate that DenseNet121 achieves faster training and inference compared with MaxViT-Tiny, highlighting a favorable accuracy–efficiency trade-off for screening-oriented deployments where throughput and resource constraints are critical.

In parallel with architectural comparisons, recent peer-reviewed literature has explored complementary methodological strategies that may further improve blood smear-based analysis under data and annotation constraints. Domain adaptation approaches have been investigated to mitigate distribution shifts caused by variations in staining protocols, microscope optics, and acquisition devices, thereby improving robustness and cross-institutional generalizability in medical imaging applications [26]. Such methods are particularly relevant for peripheral blood smear analysis, where visual appearance is highly sensitive to laboratory-specific preparation and imaging conditions. Multiple instance learning (MIL) frameworks have also gained increasing attention in computational pathology by enabling image-level supervision while implicitly modeling heterogeneous cellular content within a field of view, which aligns well with blood smear analysis where pathological patterns may be driven by a small subset of abnormal cells [27]. In addition, weakly supervised cell-level approaches using sparse or indirect annotations have been proposed to support localization and segmentation without requiring exhaustive manual labeling [28]. Beyond learning-based paradigms, complementary image analysis techniques—such as color-channel-aware edge detection—have been shown to enhance morphological contrast in cytological images and may serve as useful preprocessing or hybrid components in future SCD screening pipelines [29].

Error analysis further highlighted screening-relevant behavior across models, with false-positive errors (Negative → Positive) dominating the misclassification patterns for all Top-5 architectures. Several misclassified samples were shared across multiple models, as confirmed by image-hash-based overlap analysis, suggesting that these errors arise primarily from intrinsic dataset challenges such as overlapping cells, staining artifacts, or borderline morphologies. From a screening perspective, this conservative bias favors sensitivity but increases follow-up testing burden.

These error patterns can be further explained by the interaction between intrinsic morphological ambiguity in peripheral blood smear images and the inductive biases of the evaluated architectures. Overlapping erythrocytes, staining artifacts, and borderline cell morphologies introduce non-pathological visual cues that may resemble sickling at the image level, predisposing models to false-positive predictions. DenseNet121 appears comparatively robust to such ambiguity due to its dense connectivity, which promotes feature reuse and emphasizes localized morphological cues while reducing reliance on spurious background information. In contrast, lightweight architectures trade representational capacity for efficiency, limiting discrimination in borderline cases. Hybrid architectures such as MaxViT-Tiny integrate both local convolutional features and global contextual attention; while this contributes to strong overall performance, it may also increase sensitivity to contextual artifacts, explaining the observed tendency toward high-confidence false positives in ambiguous samples.

Screening-oriented evaluation reinforced these observations. Threshold sweep analysis demonstrated clear sensitivity–specificity trade-offs, allowing operating points to be adjusted according to screening priorities. Calibration analysis indicated generally reasonable probabilistic behavior, with mild overconfidence in certain probability ranges. These findings support confidence-aware decision support strategies, in which high-confidence positives may trigger confirmatory testing while lower-confidence cases are flagged for manual review.

Finally, qualitative explainability analysis provided insights into architectural behavior. CNN-based models tended to focus on localized RBC regions associated with morphological abnormalities, whereas hybrid models such as MaxViT-Tiny appeared to integrate both local cellular features and broader spatial context. These observations suggest, rather than confirm, differences in feature utilization across architectural families and should be interpreted as exploratory in the absence of quantitative localization validation.

No separate ablation study was conducted for data augmentation or resampling strategies, as the primary objective of this work was controlled architectural comparison under a fixed and clinically motivated training protocol. Augmentations were intentionally conservative and applied uniformly across all models, and resampling was restricted to the training set to preserve real-world screening distributions in validation and testing. In addition, the current study relies on a publicly available blood smear dataset collected outside Saudi Arabia, which lacks patient-level clinical metadata and expert-validated annotations. To address these inherent limitations of publicly available datasets, ongoing and future work focuses on collecting locally curated Saudi blood smear samples with associated clinical information and expert hematologist review, enabling more comprehensive evaluation, clinically grounded validation, and systematic analysis beyond the scope of this controlled benchmark.

Overall, this work demonstrates that architecture choice influences robustness, error characteristics, and screening suitability in blood smear-based SCD classification. Rather than claiming clinical readiness, the study emphasizes methodological rigor, uncertainty-aware evaluation, and controlled comparison as necessary steps toward responsible AI-assisted screening in hematology.

Within the context of national health transformation, AI-assisted analysis of peripheral blood smears may support the objectives outlined in Saudi Arabia’s Vision 2030 and the Health Sector Transformation Program, particularly in strengthening early detection pathways, improving screening efficiency, and enabling scalable digital decision-support tools in hematology services. While the present study does not claim clinical readiness, its controlled and leakage-safe evaluation framework provides methodological insights that may inform future development and validation efforts aligned with national digital health initiatives [19,20].

## 6. Limitations and Future Works

This study has several limitations that should be considered when interpreting the results. First, the use of a publicly available Kaggle dataset may not fully capture real-world clinical distribution shifts arising from differences in staining protocols, microscope optics, image acquisition devices, and patient demographics. As a result, models trained under these conditions may exhibit degraded performance when deployed across institutions without appropriate domain adaptation or recalibration.

Second, although bounding-box-annotated images were explicitly excluded to mitigate shortcut learning, reliance on image-level supervision still poses a risk of models exploiting non-biological cues such as background texture, staining artifacts, or slide-level characteristics rather than true cellular morphology.

Third, patient-level separation and external validation could not be enforced due to the absence of patient identifiers in the public dataset, representing a significant barrier to direct clinical translation and highlighting the need for future validation on multi-center, patient-linked datasets before screening deployment.

Future work should extend explainability analysis and error mitigation strategies by incorporating advanced XAI techniques (e.g., attention attribution, layer-wise relevance propagation) alongside cost-sensitive and ensemble learning approaches to better understand and reduce false-positive errors in screening-oriented settings.

Accordingly, future extensions should include more diverse datasets from multiple populations and clinical environments, as well as broader image acquisition and staining variations where feasible, to improve model robustness and generalizability.

Although the current work includes metric-level calibration and confidence interval analysis, future research should explore integrated uncertainty estimation at the inference level and model calibration strategies demonstrated to improve reliability in medical imaging classifiers [30].

## 7. Conclusions

This study presented a controlled, leakage-safe benchmark for SCD classification from peripheral blood smear images, explicitly designed to isolate architectural effects under data-constrained conditions. In contrast to prior studies that typically evaluate a small number of models under heterogeneous experimental settings, this work systematically compared 11 ImageNet-pretrained architectures spanning CNN-based, transformer-based, and hybrid CNN–Transformer paradigms using a unified preprocessing, training, and evaluation protocol.

A central contribution of this study lies in its explicit, leakage-safe evaluation design, which incorporates duplicate-aware, group-based data splitting and repeated splits to assess robustness. Performance assessment extended beyond point estimates by integrating statistical uncertainty analysis, paired significance testing, structured error analysis, calibration assessment, and qualitative explainability (XAI). This combined evaluation framework provides a more reliable and interpretable characterization of model behavior than accuracy-only comparisons commonly reported in the literature.

Under data-limited conditions, convolutional and hybrid architectures demonstrated more stable and robust performance than pure transformer models, although differences among the top-performing models were modest and largely overlapping within statistical uncertainty. Error analysis revealed a consistent dominance of false-positive predictions across models, underscoring the importance of threshold selection, calibration, and uncertainty-aware interpretation in screening-oriented workflows. Qualitative XAI analysis suggested that CNN-based models tend to focus on localized red blood cell morphology, whereas hybrid architectures integrate both local cellular features and broader contextual information; these observations are exploratory and not intended as validated localization claims.

Importantly, this study does not claim clinical readiness. The use of a public dataset without patient-level identifiers, the absence of external or multi-center validation, and reliance on image-level supervision limit direct clinical generalizability. These factors are acknowledged as barriers to translation rather than shortcomings of the comparative methodology.

In summary, the primary contribution of this work is methodological rather than clinical. By combining a controlled multi-architecture comparison with leakage-safe evaluation and multi-dimensional analysis of performance, errors, uncertainty, and explainability, this study provides a rigorous reference framework for future AI-based blood smear screening research. Future work should extend this framework toward externally validated, patient-level datasets, integrate explicit red blood cell segmentation for cell-level analysis and interpretability, and incorporate uncertainty-aware decision support before any real-world deployment.

## Figures and Tables

**Table 1:** Summary of prior AI-based SCD blood smear studies and reported performance.

Study (Year)	Task Level	Dataset/Source	N (Images/Smears/Patients)	Split Protocol	Preprocessing	Model/Pipeline	Reported Metrics	Key Limitations
Manescu et al. (2020) [9]	Sample-level (weakly supervised)	Digitized thin blood smears (single-center)	~140 blood smear samples (multi-FOV; 1500–4000 RBCs per smear)	Single-center split	Field-of-view aggregation; no cell-level labels	CNN + MOFF feature fusion	Accuracy ≈ 91%	Single-center; no uncertainty or calibration
Sadafi et al. (2020) [10]	Sample-level (cell-based MIL)	Bright-field microscopy blood smears	208 images	Single-center split	Cell detection + feature aggregation	Mask R-CNN + attention-based MIL	Accuracy ≈ 79%	Detection-dependent; no CI or calibration
Goswami et al. (2024) [22]	Image-level	blood smears captured from Kasturba Medical College (KMC)	191 images	Single train/test split	Resizing; basic augmentation	CNNs (GoogLeNet, ResNet18, ResNet50)	Accuracy ≈ 94.9–97%	No uncertainty analysis; no calibration
This study	Image-level	Public Kaggle PBS dataset	569 images	Duplicate-aware, group-based repeated splits	Resize; ImageNet normalization; conservative augmentation (flip, ±10° rotation)	Controlled multi-architecture benchmark under identical training budget (CNN/ViT/Swin/MaxViT)	MaxViT Accuracy, Macro-F1, ≈95% CI, McNemar test, ROC/PR analysis, calibration	No external or multi-center validation

**Table 2:** Technical characteristics and class composition of the PBS image dataset used in this study.

Characteristic	Description
Dataset name	Sickle Cell Disease Dataset (Kaggle)
Imaging modality	Bright-field light microscopy
Sample type	PBS images
Classification task	Binary classification (SCD vs. non-SCD)
Positive class	SCD (sickled erythrocytes)
Negative class	Non-sickled (normal) erythrocytes
Total number of images	569
Number of Positive images	422 (74.2%)
Number of Negative images	147 (25.8%)
Class imbalance ratio	~2.9:1 (Positive:Negative)
Image format	JPEG/PNG
Annotation type	Image-level labels (bounding-box annotated positives available but excluded from training)
Patient identifiers	None (fully anonymized)
Ethical approval	Not required (public, anonymized dataset)
Source availability	Publicly available on Kaggle: URL: https://www.kaggle.com/datasets/florencetushabe/sickle-cell-disease-dataset (accessed on 1 December 2025)

**Table 3:** Mean macro-averaged performance across repeated group-aware splits (mean ± SD).

Model	Architecture	Accuracy (Mean ± SD)	Macro-Precision (Mean ± SD)	Macro-Recall (Mean ± SD)	Macro-F1 (Mean ± SD)
MaxViT-Tiny	Hybrid CNN–Transformer	0.935 ± 0.026	0.921 ± 0.036	0.911 ± 0.033	0.915 ± 0.030
DenseNet121	CNN	0.933 ± 0.021	0.924 ± 0.051	0.907 ± 0.061	0.912 ± 0.024
InceptionV3	CNN	0.933 ± 0.022	0.921 ± 0.054	0.909 ± 0.070	0.912 ± 0.032
MobileNetV2	CNN	0.933 ± 0.021	0.914 ± 0.056	0.917 ± 0.066	0.913 ± 0.032
ResNet50	CNN	0.931 ± 0.023	0.919 ± 0.033	0.905 ± 0.067	0.909 ± 0.033
ResNet18	CNN	0.930 ± 0.032	0.914 ± 0.046	0.907 ± 0.058	0.910 ± 0.041
Swin-B	Hierarchical Transformer	0.928 ± 0.025	0.911 ± 0.040	0.908 ± 0.055	0.908 ± 0.028
VGG19	CNN	0.926 ± 0.030	0.915 ± 0.052	0.898 ± 0.078	0.904 ± 0.043
VGG16	CNN	0.912 ± 0.029	0.891 ± 0.033	0.878 ± 0.067	0.882 ± 0.046
EfficientNet-B0	CNN	0.912 ± 0.042	0.879 ± 0.052	0.910 ± 0.051	0.893 ± 0.045
ViT-B/16	Transformer	0.910 ± 0.022	0.886 ± 0.044	0.883 ± 0.057	0.885 ± 0.034

**Table 4:** Comparative performance of evaluated deep learning architectures on the held-out test set (single split, *n* = 86).

Model	Architecture Type	Accuracy	Precision (Macro) *	Recall (Macro) *	F1-Score (Macro) *
MaxViT-Tiny	Hybrid CNN–Transformer	0.942	0.960	0.914	0.937
InceptionV3	CNN	0.930	0.952	0.897	0.924
ResNet18	CNN	0.930	0.952	0.897	0.924
MobileNetV2	CNN	0.930	0.939	0.905	0.921
Swin-B	Hierarchical Transformer	0.919	0.920	0.896	0.908
VGG16	CNN	0.919	0.920	0.896	0.908
ResNet50	CNN	0.919	0.920	0.896	0.908
DenseNet121	CNN	0.919	0.945	0.879	0.910
VGG19	CNN	0.907	0.902	0.887	0.894
EfficientNet-B0	CNN	0.907	0.890	0.912	0.901
ViT-B/16	Transformer	0.756	0.728	0.706	0.714

* Macro-averaged metrics were computed by averaging class-wise precision, recall, and F1-scores across positive and negative classes.

**Table 5:** Bootstrap 95% Confidence Intervals for Top-5 Models (Reference Split).

Model	Accuracy (95% CI)	Macro-F1 (95% CI)	Sensitivity (95% CI)	Specificity (95% CI)	FPR (95% CI)
MaxViT-Tiny	0.942 [0.895–0.988]	0.933 [0.866–0.986]	0.982 [0.941–1.000]	0.862 [0.727–0.968]	0.138 [0.032–0.273]
DenseNet121	0.930 [0.872–0.977]	0.919 [0.850–0.974]	0.982 [0.944–1.000]	0.828 [0.679–0.957]	0.172 [0.043–0.321]
MobileNetV2	0.919 [0.860–0.977]	0.908 [0.839–0.969]	0.947 [0.880–1.000]	0.862 [0.733–0.969]	0.138 [0.031–0.267]
InceptionV3	0.907 [0.837–0.965]	0.890 [0.805–0.955]	0.982 [0.944–1.000]	0.759 [0.583–0.906]	0.241 [0.094–0.417]
ResNet50	0.895 [0.826–0.953]	0.872 [0.786–0.944]	1.000 [1.000–1.000]	0.690 [0.517–0.857]	0.310 [0.143–0.483]

**Table 6:** Paired McNemar Exact Test Results (Top-5 Models).

Model Pair	n01 (A Correct, B Wrong)	n10 (A Wrong, B Correct)	*p*-Value	Bonferroni-Adjusted *p*
MaxViT-Tiny vs. ResNet50	5	1	0.219	1.000
DenseNet121 vs. ResNet50	5	2	0.453	1.000
MaxViT-Tiny vs. InceptionV3	6	3	0.508	1.000
MaxViT-Tiny vs. MobileNetV2	4	2	0.688	1.000
DenseNet121 vs. InceptionV3	4	2	0.688	1.000
MobileNetV2 vs. ResNet50	5	3	0.727	1.000

**Table 7:** Computational Cost and Model Complexity for the Top-5 Models.

Model	Train Cost (10 Epochs), Mean ± SD (s)	Params (M, Approx.) *	Screening Note
MaxViT-Tiny	222.44 ± 10.99	~31 M	Highest accuracy, highest cost
InceptionV3	159.00 ± 1.11	~24 M	Larger input (299 × 299)
DenseNet121	144.01 ± 2.23	~8 M	Best accuracy–efficiency trade-off
ResNet50	136.89 ± 1.30	~25 M	Moderate performance/cost
MobileNetV2	107.82 ± 1.00	~3.5 M	Fastest, resource-efficient

* All timing measurements were obtained on Apple Silicon (MPS). Values are reported as mean ± SD and are intended for relative efficiency comparison.

## Data Availability

The data presented in this study are publicly available. The Sickle Cell Disease Dataset used for analysis can be accessed via Kaggle at URL: https://www.kaggle.com/datasets/florencetushabe/sickle-cell-disease-dataset (accessed on 1 December 2025). No new data were generated during this study.

## Abbreviations

The following abbreviations are used in this manuscript:

SCD	Sickle Cell Disease
SCT	Sickle Cell Trait
RBC	Red Blood Cell
ViT	Vision Transformer
CNN	Convolutional Neural Network
AI	Artificial Intelligence
HPLC	high-performance liquid chromatography
PCR	Polymerase Chain Reaction
XAI	Explainable Artificial Intelligence
Grad-CAM	Gradient-weighted Class Activation Mapping
PBS	Peripheral Blood Smear
MOFF	Multiple Objects Feature Fusion
MIL	Multiple Instance Learning
